# Nanoparticles and photochemistry for native-like transmembrane protein footprinting

**DOI:** 10.1038/s41467-021-27588-8

**Published:** 2021-12-14

**Authors:** Jie Sun, Xiaoran Roger Liu, Shuang Li, Peng He, Weikai Li, Michael L. Gross

**Affiliations:** 1 Department of Chemistry, Washington University in St. Louis, One Brookings Drive, Box 1134, Saint Louis, MO 63130 USA; 2 Department of Biochemistry and Molecular Biophysics, Washington University School of Medicine, 660 S. Euclid Ave, Box 8231, St. Louis, MO 63110 USA

**Keywords:** Membrane proteins, Mass spectrometry, Nanoparticles, Structural biology

## Abstract

Mass spectrometry-based footprinting can probe higher order structure of soluble proteins in their native states and serve as a complement to high-resolution approaches. Traditional footprinting approaches, however, are hampered for integral membrane proteins because their transmembrane regions are not accessible to solvent, and they contain hydrophobic residues that are generally unreactive with most chemical reagents. To address this limitation, we bond photocatalytic titanium dioxide (TiO_2_) nanoparticles to a lipid bilayer. Upon laser irradiation, the nanoparticles produce local concentrations of radicals that penetrate the lipid layer, which is made permeable by a simultaneous laser-initiated Paternò–Büchi reaction. This approach achieves footprinting for integral membrane proteins in liposomes, helps locate both ligand-binding residues in a transporter and ligand-induced conformational changes, and reveals structural aspects of proteins at the flexible unbound state. Overall, this approach proves effective in intramembrane footprinting and forges a connection between material science and biology.

## Abstract (web summary)

The intrinsic flexibility of membranes proteins still poses a challenge in determining their active structure. Here the authors describe the development of a method that combines chemical footprinting and mass spectrometry to assist in determining the structure of native membrane proteins and their dynamics.

## Introduction

Integral membrane proteins (IMPs) perform key roles in cellular signaling, transport, ahesion, and catalysis, motivating their choice for 60% of drug targets^1^. Although the human genome encodes ~5500 IMPs, only ~200 unique structures have been determined to date by X-ray crystallography^2^ and cryo-EM^3^ owing to technical challenges and time demands. Furthermore, these approaches do not inform on protein dynamics and may suffer if the protein denatures once isolated from the native membranes. Thus, a significant need exists for methodology to investigate native protein structure in the membrane environment. Mass spectrometry (MS) may provide a solution^4^.

Currently, there are two major MS-based approaches to study IMPs. One is native spray conducted in the gas phase applied so far to mainly bacterial IMPs, providing ligand identification, binding stoichiometry, but no spatial information^5,6^. Another is chemical footprinting that, via proteomic analysis of a covalently labeled protein, offers sufficient spatial resolution to answer structural biology questions^7–14^. Tailored reagents can be designed to react with solvent-accessible amino acid residues to report on binding sites, conformational changes, and dynamics^15^. The most common footprinting approach for IMPs utilizes hydroxyl radicals (•OH) because they offer comprehensive coverage especially of hydrophobic residues. When applied on the fast photochemical oxidation of proteins (FPOP) platform, the •OH can react in μs to capture the protein native states and dynamics^16,17^.

Although several investigators have applied chemical footprinting approaches to IMPs^9,10,13^, most footprints are of solvent-accessible extramembrane regions, whereas the hydrophobic transmembrane (TM) regions remain largely untouched. Footprints of the TM region of a bacterial IMP in micelles were obtained by using amphiphilic carbenes over minutes of laser and chemical precursor exposure^8^. In a specialized example, trapped functional water molecules were ionized by X-rays to generate •OH that footprints nearby regions, allowing the water molecules to be located^18,19^. Unfortunately, these methods are not yet generally applicable to IMPs, especially human IMPs that are often less stable in a detergent solution^20–22^.

**Fig. 1:**
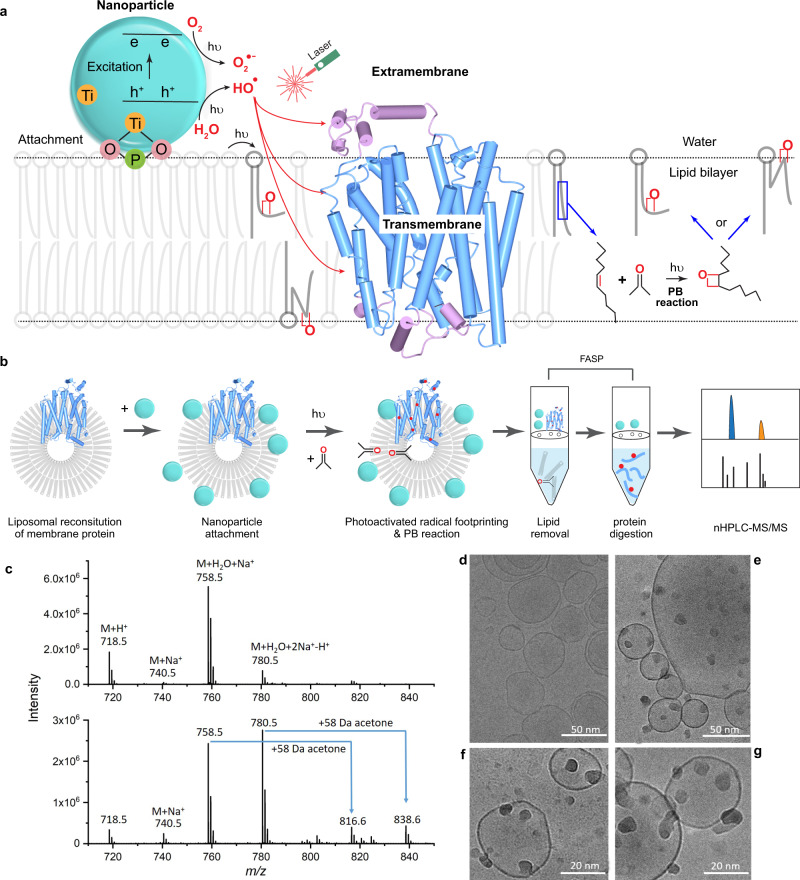
Workflow and characterization of NanoPOMP. **a** Scheme of NanoPOMP for IMP stabilized in liposome. TiO_2_ NPs are attached to the phosphate groups on the lipid bilayer. The liposome containing the IMP is irradiated with a 248 nm KrF laser, generating radicals (red dots). The PB reaction of acetone and lipid double bonds generates kinks in bilayer by a reaction mechanism shown on the right. **b** Typical workflow for the NanoPOMP experiment. (Although the protein is positioned in both orientations with equal probability in a liposome, we show only one orientation as an example.) **c** Upper: zoomed ESI mass spectrum of POPE and acetone/H_2_O; lower: ESI mass spectrum of POPE and acetone/H_2_O induced by NanoPOMP laser. Source data are provided as a Source Data file. **d** Cryo-EM image of liposome. **e**–**g** Cryo-EM images of TiO_2_ NPs (dark small spheres) attached to the liposome (large spheres), demonstrating liposome stability upon adsorption of NPs onto the surface. Higher magnification is shown in (**f**, **g**) for different sections of a cryo-EM grid. Experiments of **d**–**g** are repeated twice independently with similiar results.

Two significant challenges must be faced to elevate footprinting for IMPs: (i) generating broad coverage over hydrophobic TM regions to afford sufficient resolution for answering structural questions; and (ii) footprinting IMPs in their near-native state. To meet these two challenges, we developed and report here a strategy (Fig. 1a) that utilizes photocatalytic membrane-associated titanium dioxide nanoparticles (TiO_2_ NPs) to produce, upon laser irradiation, high local concentrations of free radicals. The irradiation simultaneously initiates a Paternò–Büchi (PB) reaction with the phospholipids comprising the liposome, to perturb the lipid region and allow the radicals to penetrate rapidly the lipid bilayer and footprint the IMP. This strategy, named NanoPOMP (NP-promoted photochemical oxidation of membrane proteins), yields high coverage in the TM region of bacterial vitamin K epoxide reductase (VKOR) and enables footprinting the conformational changes of human glucose transporter 1 (hGLUT1).

## Results

### Design and features of NanoPOMP

The design of NanoPOMP is shown in Fig. 1a. NanoPOMP has three features. First, TiO_2_ NPs^23–25^ generate reactive oxygen radicals (e.g., •OH, O_2_^•−^) to be used in a unique way for IMP footprinting. The UV laser provides sufficient energy to excite electrons to the conduction band, leaving holes (h^+^) on the valence band. Reactions of h^+^ with surface-bound H_2_O generate •OH. Electrons in the conduction band reduce O_2_ to superoxide. The h^+^ can also accept electrons directly from molecules adsorbed onto the TiO_2_ surface^26,27^.

Second, it is well-known that some metal oxides, such as TiO_2_, can reversibly bind with phosphate groups with high specificity via coordination chemistry^28^. Owing to the specific and reversible interactions with the phospholipids and other interactions including van der Waal forces, the TiO_2_ NPs attach to the surface of the liposome, which provides a near-native membrane environment for the IMP. The attached NPs generate local concentrations of •OH during laser irradiation. Considering practicality, we can remove the insoluble NPs after footprinting.

Third, a laser-initiated PB reaction increases the permeability of the lipid bilayer by a [2 + 2] cycloaddition of a double bond of the lipid with certain amount of added acetone, resulting in a fast and specific modification of the lipids^29,30^. The laser shot of the NanoPOMP platform has sufficient energy to accelerate the PB reaction. Although there are options, acetone was our first choice as the PB reagent because it is miscible with H_2_O and lipids, and is activated at 248 nm with the FPOP laser. There are three possible mechanisms that increase the permeability of the lipid bilayer: (i) acetone increases membrane fluidity and permeability, (ii) oxygen addition forms a four-membered ring structure (oxetane) by reacting with a double bond of the unsaturated lipid tail, presumably affecting the regularity and hydrophobicity of the tail, generating kinks in the bilayer, and producing defects in the membrane, and (iii) the polar oxetane group causes the modified lipids to flip toward the aqueous phase. Irrespective of mechanism, the lipid bilayer becomes more hydrophilic and permeable for hydrophilic hydroxyl radicals to access and label the transmembrane domain.

The two photochemical processes, •OH radical production and PB reaction, occur rapidly and nearly simultaneously in NanoPOMP. The free radicals are generated in the vicinity of the liposome, and the membrane permeability is increased, allowing those free radicals to access the transmembrane region.

### Characterization of the NanoPOMP system

Firstly, we showed that the PB reaction occurs under our fast footprinting condition of the NanoPOMP platform (i.e., one laser shot at a power of 23 mJ). Using a typical NanoPOMP protocol, we flowed a mixture of POPE and acetone/H_2_O and submitted the mixture to nanoPOMP, followed by characterization of the products by ESI MS. Although the POPE concentration was 32 μM (to avoid contaminating the mass spectrometer), a concentration that is much lower than that of POPE when forming liposomes for NanoPOMP (~28 mM), we reasoned that if the PB reaction occurs at this concentration, it should also take place under the higher concentrations used in the NanoPOMP. The product peaks corresponding to acetone addition are clearly seen (Fig. 1c), showing that the PB reaction occurs upon activation by a few ns laser pulse. These conditions are appropriate for the PB reaction to modify the unsaturated lipid tail and to make the membrane more permeable.

Another important question is whether the attachment of TiO_2_ NPs perturbs the liposome, causing it to deform or even fall apart. We used electron microscopy at cryogenic temperature (Cryo-EM) to visualize the liposome before and after adsorption of TiO_2_ NPs (Fig. 1d–g) onto the surface because Cryo-EM can better maintain liposome structure compared with other methods. A control (Fig. 1d) shows the intact liposome. After incubation with NPs (1 h, room temperature (RT)), the sample was frozen immediately and imaged instantly (Fig. 1e–g). We clearly see that TiO_2_ NPs are attached securely to the surface and that the liposomes remain circular and intact. Figure 1e–g (f and g with higher magnification) show, at least at this resolution, that the liposome edge is intact, suggesting that addition of TiO_2_ NPs does not perturb the liposome structure.

In addition to the imaging of the TiO_2_ NPs-POPE/POPG liposome system, previous studies further support the view that the NPs used here do not significantly affect the liposome structure. The liposome/NP interaction includes van der Waals, double layer, hydration, hydrophobic, thermal undulation, and protrusion forces^31^. The size of the NPs play a role in the interaction^32–34^, for example, 5 nm AuNPs (the particle diameter used here) have the smallest influence on the membrane compared to larger sizes^32^. In another case, suspensions of SiO_2_ NPs and DPPC liposomes are stable for months for mixtures of 50 nm liposomes interacting with nominal 4–6 nm SiO_2_ NPs^35^. Charge is another parameter that influences the interaction. Studies showed that the attachment of charged NPs improves liposome stability by increasing the absolute zeta potential of the dispersion and by introducing charges on the liposome surface^35–37^. Furthermore, the coordination chemistry between TiO_2_ and the phosphate group allows TiO_2_ to bind reversibly with liposomes with high specificity^28^, imparting stabilizing properties not found for other NPs. For example, the interaction of TiO_2_ NP with DOPC/DOCPe/DOPS liposomes via coordination is found to be more important than charge^38^. Other research showed that TiO_2_ NPs stabilize a DPPC liposome even under the lengthy conditions needed to reach adsorption equilibrium of TiO_2_ with the vesicles^39^.

### Performance optimization of NanoPOMP on bacterial VKOR, a model IMP

The second question we asked is whether the combination of a PB reaction initiated simultaneously by the laser pulse that generate radicals affords membrane protein footprinting. We used bacterial VKOR (PDB ID: 3KP9) to answer this question. VKOR is an integral membrane enzyme that couples vitamin K reduction with disulfide-bond formation, to sustain oxidative protein folding in prokaryotes and support blood coagulation in humans^40^. We optimized a workflow (Fig. 1b) following the footprinting (Supplementary Fig. 1) to separate the TiO_2_ and to remove lipids, salts, and any residual TiO_2_ by filter-aided sample preparation (FASP^41^, Supplementary Fig. 2). The steps are intended to minimize contamination of the nHPLC column and the mass spectrometer with lipids. With this workflow, we could isolate the VKOR and analyze it with high (~100%) sequence coverage (Supplementary Fig. 3). The TiO_2_ we used here has high UV absorption (Supplementary Fig. 4) to afford sufficient quantum yield and thus catalytic reactivity to produce abundant radicals.

**Fig. 2:**
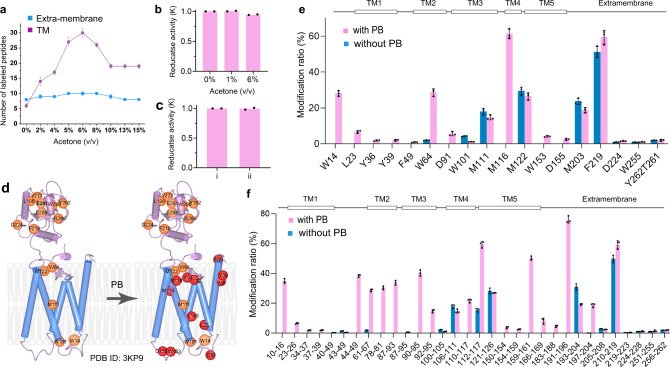
NanoPOMP performance on VKOR. **a** The number of labeled peptides for the extra-membrane and TM regions of VKOR (PDB ID: 3KP9^40^) as a function of the amount of acetone (blue-extra-membrane; purple-TM) (*n* = 3 independent experiments per group, data are presented as mean values ± SD). **b** Enzyme activity of VKOR in liposome in presence of different amount of acetone (*n* = 2 independent experiments per group, data are presented as mean values and dots represent the two corresponding individual repeats). **c** Enzyme activity of VKOR in liposome (i) without TiO_2_ NPs, and (ii) with TiO_2_ NPs. *n* = 2 independent experiments per group, data are presented as mean values and dots represent the two corresponding individual repeats. **d** Cartoon diagram showing sites labeled by NanoPOMP. Red circles represent modified sites in the TM with PB reaction, and orange circles represent those without PB. **e** Residue-level modification ratios upon irradiation of TiO_2_ NPs with and without the PB reaction (purple-with PB; blue-without PB). *n* = 3 independent experiments per group, data are presented as mean values ± SD and dots represent the corresponding individual repeats. **f** Modification ratios at peptide level (purple-with PB; blue-without PB). *n* = 3 independent experiments per group, data are presented as mean values ± SD and dots represent the corresponding individual repeats. Source data are provided as a Source Data file.

Next, we optimized the amount of PB reactant (acetone) by testing several levels from 0 to 15% (v/v) (Fig. 2a). The number of footprinted extramembrane domain peptides remained nearly constant, whereas greater amounts of acetone presumably increase the lipid-membrane perturbation; the acetone amount that affords the highest yield of TM-domain footprints is 6% in the aqueous phase. The peptide-level results with PB and without PB clearly demonstrate that the TM regions undergo footprinting following membrane perturbation (Fig. 2f). The residue-level results (Fig. 2d, e) demonstrate, upon bilayer perturbation, that footprints are produced over almost the entire VKOR held within the liposome. Residues Y, W, F, M, T, D, and L become modified to different extents (examples of EIC and product-ion (MS^2^) spectra are in Supplementary Figs. 5–14).

### Effect of PB Reagent and TiO_2_ NPS on IMP’s structure and activity

A potential issue to be faced is the effect of the added acetone on the protein structure. We obtained a circular dichroism spectrum of VKOR with 6% acetone and found no significant difference (Supplementary Fig. 15), indicating that secondary structure is minimally disrupted if at all. To provide further evidence that acetone is not detrimental to the tertiary structure and enzyme function, we used VKOR enzymatic activity assays (Fig. 2b). With 1% acetone, VKOR activity increased slightly probably because the substrate (vitamin K) dissolved better. With 6% acetone, VKOR showed essentially no change in its enzymatic activity compared with no acetone. We conclude the amount of acetone used in this NanoPOMP protocol is in the “safe zone” to maintain VKOR structure and enzymatic activity. We also tested the enzyme activity of VKOR (Fig. 2c, Supplementary Fig. 16) in liposome (i) and VKOR in liposome incubated with TiO_2_ NPs (ii). The results show that binding TiO_2_ NPs does not significantly affect the enzymatic activity, suggesting the IMP is stabilized in the liposome.

### NanoPOMP mapping of a human IMP

**Fig. 3:**
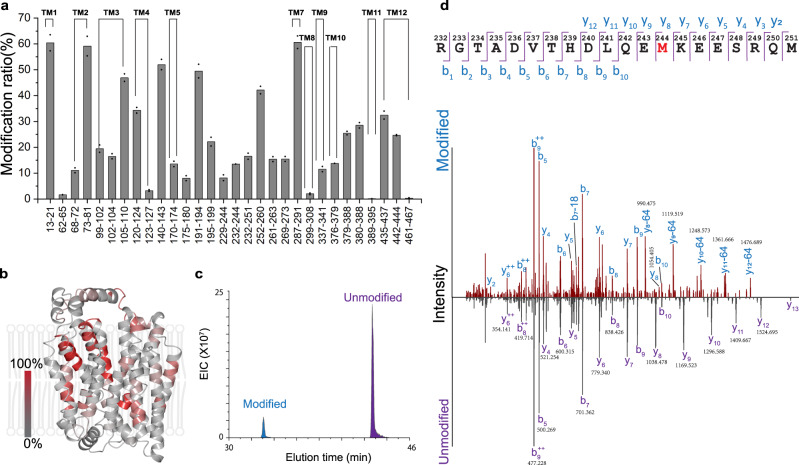
NanoPOMP of hGLUT1 in liposome. **a** Modification ratios at the peptide level. *n* = 2 independent experiments were repeated, two technical replicates (injections) were conducted for each individual sample. Data are presented as mean values of two biological repeats and dots represent the two corresponding biologically repeats. **b** Heat map (shaded in red) of the modification extents at the peptide level. **c** Extracted ion chromatogram of modified and unmodified representative peptide 232–251. **d** CID product-ion (MS^2^) spectra of modified and unmodified peptide 232–251. Source data are provided as a Source Data file.

Then, we applied NanoPOMP to another IMP, hGLUT1, which is ubiquitous in erythrocytes and blood–tissue barriers in humans and is upregulated in cancer cells. Following the workflow established for VKOR, we achieved ~100% sequence coverage of hGLUT1 (Supplementary Fig. 17). In separate experiments, TiO_2_-FPOP mainly footprints the extra-membrane regions (Supplementary Table 3 summarizes the results of MS analysis showing EICs, modified ratios, modified peptides, and residue-level information). The NanoPOMP protocol, however, led to footprinting of both the transmembrane and extramembrane regions of hGLUT1 (Fig. 3a, b). We found that 11 of 12 transmembrane helices (TM) were footprinted. For TM2, 3, 4, and 12, more than one site became modified (Fig. 3c shows an example of the EIC of peptide 232–251). By comparing the product-ion (MS^2^) spectra of the two peptides (Fig. 3d), we identified the footprinting site to be M244. (Detailed information, including mass spectra, EICs, identities of modified peptides and residues, and modification extents for all other footprinted peptides are in Supplementary Table 4. Examples of EICs, mass spectra, and MS^2^ spectra of labeled and unlabeled peptides are in Supplementary Figs. 18–25).

This protocol may be useful to probe the interaction of IMPs with small-molecule drugs where assessing conformational change of a protein upon interaction is a major challenge. To test whether NanoPOMP can provide a meaningful footprint of the conformational changes induced by ligand binding, we expanded our study to include interactions of hGLUT1 with *D*-maltose (Mal) and cytochalasin B (CyB). Mal binding affords an outward-open whereas Cyb binding gives an inward-open conformation^42–44^, a typical situation with membrane transporters. The structure of hGLUT1 in its inward-open conformation is known^42,45^, whereas its outward-open conformation can be reliably modeled from bovine GLUT5 structure because their structures are highly similar; in their inward-facing conformation, the root mean square deviation RMSD (Cα–Cα) between their structures (PDB 4PYP and 4YB9) is only 1.4 Å^46^.

To test the capability of NanoPOMP to follow the conformational changes, we used it in a typical differential footprinting experiment to probe the structural differences of the two hGLUT1 conformations. In this way, we address changes in higher order structure at the peptide/residue level by comparison of footprinting the two ligand-bound states^14,47^. We then compare the footprinting results with the changes in solvent accessible surface area (SASA) that are calculated from the two structures. Changes in SASA can evaluate the extent of protection/exposure (i.e., a conformational change leading to an increase in SASA causes increased modification whereas ligand binding decreases the SASA of critical binding residues and lowers their modification extents (protection)). The design acknowledges that footprinting is usually a differential experiment in which results are compared with the ligand absent or present^47^.

**Fig. 4:**
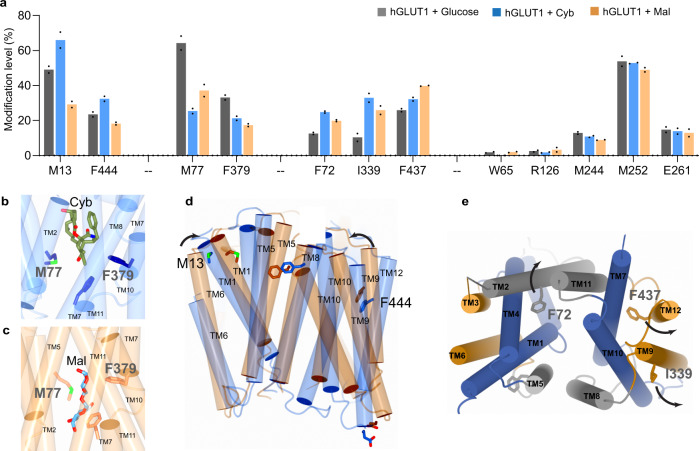
NanoPOMP helps to reveal conformational change and ligands binding. **a** Modification extents at the residue level for three binding states of hGLUT1 (grey-hGlut1 + Glucose; blue-hGlut1 + Cyb; orange-hGlut1 + Mal). *n* = 2 independent experiments were repeated, two technical replicates (injections) were conducted for each individual sample. Data are presented as mean values of two biological repeats and dots represent the two corresponding biologically repeats. Source data are provided as a Source Data file. **b**, **c** Residues interacting with CyB and Mal as identified by NanoPOMP. **d** Residues reporting conformational change (indicated by arrows) from the Mal-binding state to CyB-binding state. **e** Residues reporting conformational changes from unbound state to CyB-/Mal-binding state.

The fraction modified at the residue level (Fig. 4a) of the Mal/CyB bound vs. unbound states can be classified in four groups owing to different underlying mechanisms. The first group of residues, M13 and F444, shows modification increases upon binding to CyB and decreases when binding to Mal. These sites are undergoing significant conformational change during the rocker-switch motion of the transporter between inward- and outward-facing conformations. Comparing the two structures in Fig. 4d (CyB-binding and modeled Mal-binding structure), we conclude the helices bearing M13 and F444 move outward for CyB binding, opening that region for modification, and these conclusions are consistent with the modification level changes determined by NanoPOMP. Moreover, the calculated SASA difference of CyB-binding vs. Mal-binding shows an increase for an inward- and a decrease for the outward-facing conformations (Supplementary Fig. 26), confirming again that MS-based footprinting reflects ligand-induced conformational changes, in this case, of a human IMP.

Having demonstrated that NanoPOMP is capable of revealing the conformational change induced by ligand binding, we utilized NanoPOMP to identify the transmembrane residues that interact with the two ligands, CyB and Mal. The second group of residues (Fig. 4a, M77 and F379) show decreased modification upon binding with both CyB and Mal, indicating that both residues are involved in binding with CyB and Mal. In addition to the footprinting results, two other observations show that M77 and F379 are likely part of the binding pocket. (i) The distances between these ligands and the amino acid residues in the model of the outward-open (Fig. 4b) and known inward-open (Fig. 4c) structure are in the range of non-covalent interactions that are directly involved in the binding of these ligands. (ii) According to the literature, F379 from hGLUT1 has interactions with Cyb^42^. These results advance the understanding of the hGLUT1 binding sites by a MS-based method and should enable the design and optimization of more specific inhibitors for human IMP, especially when combined with outcomes of other methods (CryoEM and modeling).

Furthermore, NanoPOMP reveals structural changes upon ligand binding. In Fig. 4a, the third group of F72, I339, and F437 show modification level increases compared to the unbound state for both CyB and Mal binding, suggesting these residues are rigid or buried in the unbound state. Upon ligand binding, they may become more flexible or orient outward and become accessible to footprinting (Fig. 4e). Similar to our footprinting that clearly detects the conformational change between the two ligand-binding states, here such change of footprints, far from the ligand-binding sites, may originate from conformational exposure induced upon ligand binding to the apo protein. Notably, the crystal structure of hGLUT1 in its unbound state cannot be obtained without stabilizing mutations owing to the intrinsic flexibility required for the protein to execute its transporter function^48^. Thus, utilization of NanoPOMP provides the first insight to understand the unbound state and free motion of hGLUT1.

### NanoPOMP with isopropanol

As a control experiment to verify the importance of the PB reaction, we compared the acetone results with those of a similar organic solvent, isopropanol (IPA), that cannot participate in a PB reaction. We incorporated several amounts of IPA (2%, 4%, and 6%) with hGlut1-liposome and compared the results with the acetone/hGlut1 system. IPA does increase the labeling coverage for the transmembrane domain of hGLUT1 but at lower efficiency than produced with acetone. Although IPA at 6% gives the highest yield (Supplementary Table 5) and footprints TM2, 6, 8, 10, and 12, almost all TM regions are footprinted with 6% acetone following the PB reaction (Fig. 3a). That addition of IPA increases footprinting may be because, as an organic solvent, it increases the liposome permeability. Based on these results, we reiterate that NanoPOMP promotes footprinting primiarly because of the PB reaction.

## Discussion

Overall, NanoPOMP is suitable for membrane protein footprinting. The approach makes use of the same platform as FPOP without the requirement of additional instrumentation. The likely reactive species in TiO_2_ photocatalysis are the holes on the TiO_2_ surface, hydroxyl radicals, superoxide ions, and singlet oxygen, in addition to trace amounts of H_2_O_2_ and O_2_. The rate constant for reaction of the hydroxyl radical, however, is significantly larger than those of superoxide ion or singlet oxygen^24^. Moreover, the oxidation of a protein is more likely to occur by hydroxyl radicals than by surface holes of TiO_2_, given that the protein is not attached directly to the surface of TiO_2_. It was also suggested that molecules need to be adsorbed onto the TiO_2_ surface to react with the short-lived singlet oxygen (lifetime 2–3 μs), so it is less likely that singlet oxygen oxidizes the protein^49,50^. Hence, in NanoPOMP, hydroxyl radicals likely play the major role in initiating the footprinting. Similar with FPOP, NanoPOMP provides a snapshot of the native protein. Although the protein may incur some structural changes due to the modification, the native state is captured by oxidation of the protein surface, and any modification-induced structural changes that occur later are unimportant. Further, NanoPOMP produces radicals in a localized manner at the surface of the liposomes. In addition, the NanoPOMP process does not require a conventional scavenger (e.g., histidine for soluble proteins) because such a scavenger likely would not enter the lipid bilayer given its larger size and its hydrophilicity. Besides, the generated hydroxyl radicals upon laser irradiation are admitted to the lipid bilayer and react with the membrane protein, whereas the excess radicals may be scavenged by reacting with double bonds of the lipids, for which they generate lipid peroxide (LOOH) as final product. In other words, the scavenging effect of lipids on the inside radicals may be larger than conventional scavengers. 

To use NanoPOMP to probe the higher order structure of IMPs, we recommend differential experiments as are used in FPOP, HDX, and other footprinting. Such measurements compare two states of a protein (e.g., bound vs. unbound, wild-type vs. mutant). Differential experiments allow any adventitious effects that affect footprinting to cancel, leaving any differences in footprinting to represent changes in solvent accessibilities of the two states.

In summary, this approach enables footprinting of IMPs in the transmembrane regions. Even more importantly, the approach can help to identify those amino acid residues of a human IMP that interact with inhibitors, suggesting that binding of small molecules to IMPs in drug development can be determined when combined with other methods. The speed of this method may be useful when a small library of compounds must be evaluated for their interactions with a target protein. This form of footprinting also reports on conformational change of IMPs upon binding. Thus, the door is opening to undertake essential but difficult experimental tasks of viewing motions of critical amino acid residues, especially when conformational flexiblity precludes structure determination by crystallography and cryo-EM. The NanoPOMP approach may be the foundation of a streamlined strategy for structural proteomics of IMPs, with applications in small-molecule drug design, binding, and screening.

## Methods

### Preparation of VKOR/liposome system

The VKOR membrane protein was expressed and purified as a bacterial homolog^40^. The VKOR-liposome system was comprised of VKOR: lipids = 1:25 (weight:weight). POPE (300 μL of 1-palmitoyl-2-oleoyl-*sn*-glycero-3-phosphoethanolamine) in dichloromethane and 100 μL POPG (1-palmitoyl-2-oleoyl-*sn*-glycero-3-phospho-1’-rac-glycerol sodium salt) in dichloromethane were mixed in a clean glass tube (5 mL), and the solvent slowly evaporated with nitrogen gas flow. The dried lipid membrane in the glass tube was stored in a vacuum chamber for 60 min to remove completely the dichloromethane. PBS buffer (900 μL 1×, pH 7.54) and 100 μL sodium cholate hydrate solution (60 mg/mL in H_2_O) were added to the glass tube and sonicated for 30 min until the suspension was clear and transparent. VKOR protein solution (6.66 μL, 60 mg/mL) was added to the lipid solution. The protein-lipid mixture was then added to pre-balanced resin columns (Sephadex^TM^ G-50 Fine), the columns were immediately centrifuged under 1500g, and the liposome solution after centrifugation was collected. The liposome solution was then centrifuged under 110,000*g* to obtain a liposome pellet that was resuspended with 50 μL Tris buffer (1×, pH 7.54) to obtain 100 μM of VKOR theoretically.

### Preparation of hGLUT1/liposome system

The hGLUT1 protein was overexpressed in human 293S cells by using a Bacmam system^51^, and purified by cobalt affinity and size-exclusion chromatography. HEK293 GnTI—suspension cells (N-acetylglucosaminyltransferase I-negative) were purchased from ATCC (American Type Culture Collection, Manassas, USA). The hGLUT1 was incorporated into liposomes by using the following protocol. POPE (300 μL in dichloromethane) and 100 μL POPG in dichloromethane were mixed in a clean glass tube (5 mL), and the solvent slowly removed under a nitrogen gas flow. The dried lipid membrane in the tube was admitted to a vacuum chamber for 60 min to remove completely the dichloromethane. PBS buffer (900 μL, 1×, pH 7.54) and 100 μL sodium cholate hydrate (60 mg/mL in H_2_O) were added to the glass tube and sonicated for 30 min until the suspension was clear and transparent. hGLUT1 protein solution (71.4 μL, 5.6 mg/mL) was added to the lipid solution. The protein-lipid mixture was added to the pre-balanced resin columns (SephadexTM G-50 Fine), the columns immediately centrifuged at 1500*g*, and the liposome solution collected. That solution was centrifuged at 110,000*g* to obtain a liposome pellet that was resuspended with 50 μL Tris buffer (1× pH 7.54) to obtain 100 μM hGLUT1 theoretically.

### Cryo-EM imaging of liposome and liposome-TiO_2_ NPs

(1) Liposome preparation: POPE (300 μL) in dichloromethane and 100 μL POPG in dichloromethane were mixed in a clean glass tube (5 mL), and the solvent slowly evaporated with dry nitrogen gas flow. The dried lipid membrane in the glass tube was stored in a vacuum chamber for 60 min to remove completely the dichloromethane. PBS buffer (900 μL 1×, pH 7.54) and 100 μL sodium cholate hydrate solution (60 mg/mL in H_2_O) were added to the glass tube and sonicated for 30 min until the suspension was clear and transparent. The mixture was then added to pre-balanced resin columns (SephadexTM G-50 Fine), the columns were immediately centrifuged under 1500 g, and the liposome solution after centrifugation was collected. (2) Freeze–thaw sample: The sample was frozen at liquid nitrogen and thawed at room temperature three times. The samples were stored at room temperature until they were imaged by Cryo-EM. (3) On the day of imaging, the sample was extruded 21 times. One of the metal rings onto the main body of the extruder was screwed in place, and the open side placed up on a tabletop. One white piece and mesh were placed in the body of the extruder, mesh screen side up, and a few drops of 1× dialysis buffer were placed onto the mesh. Forceps were used to place a 400 nm Whatman filter onto the mesh. The other white piece was placed in the metal body of the extruder, mesh side down. Another metal ring was placed on the main body of the extruder. Some 1× dialysis buffer was drawn into one of the syringes, removing any bubbles. Both syringes were attached to the body of the extruder, and the 1× dialysis buffer was passed from side to side a few times to remove bubbles, and the process was repeated until no bubbles remain. A volume of 400 µL of 1× dialysis buffer was drawn up with the syringe not used in the previous step, air bubbles removed, and the solution passed back and forth between the two syringes 21 times. A volume of 350–400 μL of liposome sample was drawn into one of the syringes by using a disposable 18 g needle. The syringes were attached to the extruder, and the sample was passed 21 times. The extruded sample was passed into a clean tube. After 1 h incubation of NPs with liposome at RT, the sample was frozen immediately and imaged instantly. The samples were applied to glow-discharged Lacey carbon support films (electron microscopy sciences, Hatfield PA) in 3 μL drops, blotted, and imaged at 120 kV in the microscope.

### Conditions for NanoPOMP of VKOR in liposome

TiO_2_ NPs (mixed phase of pure ST-01 Anatase titanium dioxide NPs and P25 TiO_2_ NPs (1:1, wt: wt), 5 nm in diameter) were suspended in water (equivalent to 200 mM) as a stock solution just prior to use. The sample suspension (50 μL) was gently stirred in the dark for 30–60 min to allow the establishment of the adsorption–desorption equilibrium between the liposome and TiO_2_ NPs. The final concentration of TiO_2_ NPs was 20 mM. The FPOP system we used contains a laser setup and flow system (Supplementary Fig. 1)^16,47^. Specifically for NanoPOMP, the operating workflow used a 248 nm KrF excimer laser (GAM Laser Inc., Orlando, FL) to photo-excite the TiO_2_ NPs. A syringe pump was connected to a silica capillary terminating in a collection tube. A 75 μm id capillary was used to increase the utilization rate of the laser photons. After setting up the laser, the laser spot width was measured. By considering the I.D. of the capillary, the illuminated volume of a single laser shot was calculated as

1 $${{{{{\rm{Illuminated}}}}}}\,{{{{{\rm{volume}}}}}}={\left(\frac{{{{{{\rm{I}}}}}}.{{{{{\rm{D}}}}}}.\times0.001}{2}\right)}^{2}* \Pi * {{{{{\rm{spot}}}}}}\,{{{{{\rm{width}}}}}}$$

With the exclusion volume, the total volume with a single laser shot was given as

2 $${{{{{\rm{Total}}}}}}\,{{{{{\rm{volume}}}}}}=\frac{{{{{{\rm{Iluminated}}}}}}\,{{{{{\rm{volume}}}}}}}{1-{{{{{\rm{exclusion}}}}}}\,{{{{{\rm{fraction}}}}}}}$$

Together with the laser frequency (7.4 Hz), the flow rate was calculated as

3 $${{{{{\rm{Flow}}}}}}\,{{{{{\rm{rate}}}}}}={{{{{\rm{laser}}}}}}\,{{{{{\rm{frequency}}}}}}* 60* {{{{{\rm{total}}}}}}\,{{{{{\rm{volume}}}}}}$$

The exclusion volume was 15%; the laser energy was 23 mJ per pulse. The polyimide coating of the capillary was removed along a small section of the tube exterior to give a transparent window for the laser beam. The laser beam was restricted by an iris and focused by three convex lenses to pass through the transparent window. In this experiment, a 50 μL sample in each tube containing different concentrations of PB reactant (acetone) were tested. The laser pulse frequency was controlled by an external pulse generator (B&K Precision, Yorba Linda, CA). After footprinting, the samples collected in vials were centrifuged to precipitate the NPs. The supernatant and NP suspension were collected separately and submitted to FASP.

### Removal of lipids and proteolysis before MS detection

Following FPOP, the labeled VKOR and hGLUT1 samples were submitted to a FASP protocol before nHPLC-MS analysis. The FASP protocol was modified slightly on the basis of previous literature^41^. Specifically, ultrafiltration units (Microcon 30 kDa MW cut off) containing 100 μL membrane proteins (after labeling, 5 μM concentration in theory) were added to 150 μL of denaturing solution containing 8 M urea (in 0.1 M ammonium bicarbonate, pH 8.0) in the ultrafiltration units and centrifuged at 10000 g for ~ 15 min until nearly all the sample passed through the filter. Then 200 μL denaturing solution was added to the ultrafiltration unit, and the wash step was repeated two more times. This step removed the lipids and salts from the membrane protein system and denatured the proteins. The solution in the collection tubes was discarded, and 100 μL 50 mM TCEP in 0.1 M ammonium bicarbonate was added to the filter and incubated at 37 °C for 30 min. Then 100 μL 100 mM iodoacetamide (IAA) was added to the filter, and the solution incubated in the dark for 30 min, centrifuged at 10000 g until all the solution passed through the filter (1 h or more required). To that solution was added 150 μL digestion buffer (0.1 M NH_4_HCO_3_), and the solution was centrifuged at 10000 g, and the process repeated two more times. Then the digestion buffer (60 μL of 0.1 M NH_4_HCO_3_) and chymotrypsin (protein to enzyme ratio 20:1) were added to the filter and incubated overnight at 37 °C in a water bath. After digestion, the ultrafiltration units were placed in new collection tubes and centrifuged at 10,000g to collect the digested peptide solution. The fresh digestion solution (50 μL of 0.1 M NH_4_HCO_3_) was added and centrifuged to the collection tube; this step was repeated two times. Formic acid (1 μL) was added to the collected peptide solution, which was used for subsequent nHPLC-MS analysis.

### NanoPOMP of hGLUT1 in liposome with different inhibitors (Mal & CyB)

The concentration of hGLUT1-liposome was determined by using a Nanodrop (Thermo Scientific Inc., Massachusetts, USA) to be 30 μM. hGLUT1-liposome (5 μL) was diluted to a final total volume of 50 μL to give a concentration of hGLUT1-liposome that was used for PB-FPOP of 3 μM. Five groups of experiments were conducted, as listed in Supplementary Table 2. The TiO_2_ NPs were prepared as 200 mM stock solution. The final concentrations of Mal and CyB were 200 and 100 μM, respectively. All the substrates were incubated with the hGLUT1-liposome for 30 min at room temperature.

### nHPLC/MS/MS analysis

The digested protein sample (15 μL) was diluted into 35 μL water with 0.1% formic acid and centrifuged at 5000 g for 1 min. The sample solution was loaded onto a custom-packed C18 reversed-phase column (Waters Symmetry, 5 μm, 100 Å, 75 μm * 20 cm) at 4 μL/min for seperation. Gradients for HPLC were comprised of (A) water with 0.1% formic acid by volume and (B) 80% acetonitrile with 0.1% formic acid by volume for an HPLC gradient of 0–10 min 2% B, 10–90 min 2% B to 60% B, 90–100 min 2% B to 98% B, 100–105 min 90% B, 105–115 min 90% B to 2% B and equilibrate at 2% B for 5 min. A Thermo Q Exactive Plus orbitrap mass spectrometer (Thermo Scientific, Waltham, MA, USA) coupled with a Nanospray Flex source for downstream detection with 3.0 kV spray voltage at 250 °C was used. Spectra were acquired in the positive-ion mode over a 300–2000 m/z range at a mass resolving power of 70,000 for MS^1^ and 16,700 for MS^2^. Results were acquired in the data-dependent mode, where the ten most abundant ions were selected for “higher energy” collisional dissociation (HCD). Signals >+6 charge state were rejected.

### Data analysis

The nHPLC-MS/MS raw files were processed by the Byonic^TM^ software (Protein Metrics, San Carlos, CA, USA) to reveal sequence coverage (20 ppm precursor mass tolerance, 60 ppm fragmentation mass tolerance, and CID/HCD fragmentation for searching). The automatically searched results were checked manually with a custom program, Thermo Xcalibur (Thermo Scientific, Waltham, MA, USA). Residue-level modifications were assigned based on product-ion spectra and were further validated by manual inspection of the MS^2^ fragmentation. The modification ratios were calculated based on the manually selected extracted ion chromatograms (EIC) area ratios according to the following equation:

4 $${{{{{\rm{Fraction}}}}}}\,{{{{{\rm{modified}}}}}}=\frac{{\sum }^{}I_{{{{{\rm{oxidation}}}}}}}{{\sum }^{}I_{{{{{\rm{oxidation}}}}}}+{\sum }^{}I}$$

Protein structures were displayed using PYMOL 2.3.2.

### Circular dichroism spectrum of membrane protein

CD spectra were acquired on a J-815 OD spectrometer (Jasco Incorporated, Easton, Maryland, USA): 500 μL VKOR protein (2 μM) was added to the cell with or without acetone and the spectrum was acquired from 190 to 320 nm.

### VKOR enzymatic activity assay

For testing the influence of acetone, the reaction was initiated by mixing 40 μL of 1 μM VKOR proteoliposome with equal volume of PBS buffer (pH 7.54) containing 120 μM vitamin K and 12.5 mM DTT. The fluorescence of the reaction product (vitamin K hydroquinone) was measured on a SpectraMax M5 (Molecular Devices, Inc., USA). The excitation and emission wavelengths were 250 and 430 nm, respectively. For testing the effects of TiO_2_ NPs on the protein, the reaction was initiated by mixing 40 μL of 5 μM VKOR proteoliposome (without and with NPs) with equal volume of PBS buffer (pH 7.54) containing 120 μM vitamin K and 12.5 mM DTT. The fluorescence of the reaction product (vitamin K hydroquinone) was measured on a SpectraMax M5 (Molecular Devices, Inc., USA). The excitation and emission wavelengths were 250 and 430 nm, respectively.

### Model building of outward-open hGLUT1

The outward-opening conformation of GLUT1 was generated from homology modeling, by using one-to-one threading in Phyre2^52^ with the structure of the GLUT3 in the outward-opening conformation (PDB: 4ZWC)^53^ as the template.

### Reporting summary

Further information on research design is available in the Nature Research Reporting Summary linked to this article.

## Supplementary information

Supplementary Information

Reporting Summary

## Data Availability

The mass spectrometry proteomics data have been deposited in the ProteomeXchange Consortium via the PRIDE partner repository with the dataset identifier PXD027819. MS^2^ spectra generated are provided in the Supplementary Information. The structures of VKOR and GLUT proteins used in this study were previously published and are available in the Protein Data Bank (PDB) under the accession codes 3KP9, 4PYP, 4YB9, and 4ZWC. Source data are provided with this paper.

## Source data

Source Data
