# Supplementary material for: Nanoparticles and photochemistry for native-like transmembrane protein footprinting
Source: Nat Commun. 2021 Dec 14;12:7270. doi: 10.1038/s41467-021-27588-8 (PMC8671412; doi:10.1038/s41467-021-27588-8)
Supplement: Supplementary file 1 — Supplementary Information [file 41467_2021_27588_MOESM1_ESM.pdf]

Supplementary Information

Nanoparticles and Photochemistry for Native-like Transmembrane Protein Footprinting

Jie Sun[1], Xiaoran Roger Liu[1], Shuang Li[2], Peng He[2], Weikai Li* [2] and Michael L. Gross* [2]

[1] Department of Chemistry, Washington University in St. Louis, One Brookings Drive, Box 1134, Saint Louis, Missouri 63130, E-mail: mgross@wustl.edu

[2] Department of Biochemistry and Molecular Biophysics, Washington University School of Medicine, 660 S. Euclid Ave, Box 8231, St. Louis, MO 63110 USA, Email: weikai@wustl.edu

Table of Contents

1. **Supplementary Figures**

Supplementary Figure 1. FPOP flow system
Supplementary Figure 2. Workflow for filter-aided sample preparation (FASP)
Supplementary Figure 3. Mass spectrometry sequence coverage of VKOR in bottom-up proteomics
Supplementary Figure 4. UV-Vis spectrum of TiO_2 nanoparticle suspension
Supplementary Figure 5-14 EIC, MS and MS^2 of unmodified and representative modified peptides from VKOR
Supplementary Figure 15 Circular dichroism spectrum of membrane protein VKOR
Supplementary Figure 16 VKOR enzymatic activity assay
Supplementary Figure 17 Mass spectrometry sequence coverage of hGLUT1 in bottom up proteomics
Supplementary Figure 18-25 EIC, MS and MS^2 of unmodified and representative modified peptides from hGLUT1
Supplementary Figure 26 SASA change upon ligand binding of hGLUT1

2. **Supplementary Tables**

Supplementary Table 1 Optimization of different conditions for NanoPOMP
Supplementary Table 2 NanoPOMP experiments of hGLUT1 in liposome
Supplementary Table 3 TiO_2-FPOP of hGLUT1
Supplementary Table 4 NanoPOMP of hGLUT1
Supplementary Table 5 Test of IPA on hGLUT1

1. Supplementary Figures

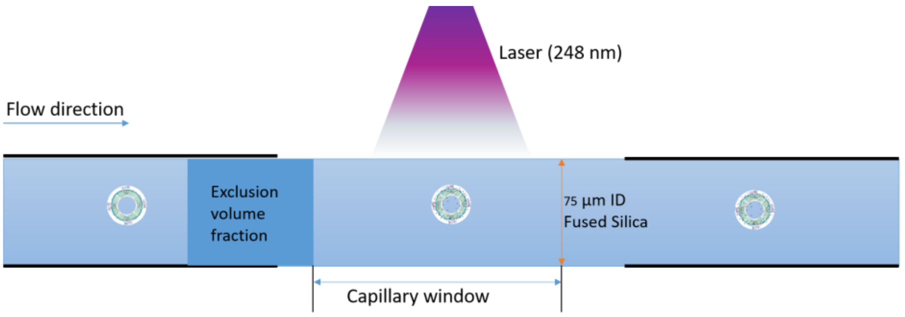

Supplementary Figure 1. NanoPOMP flow system.

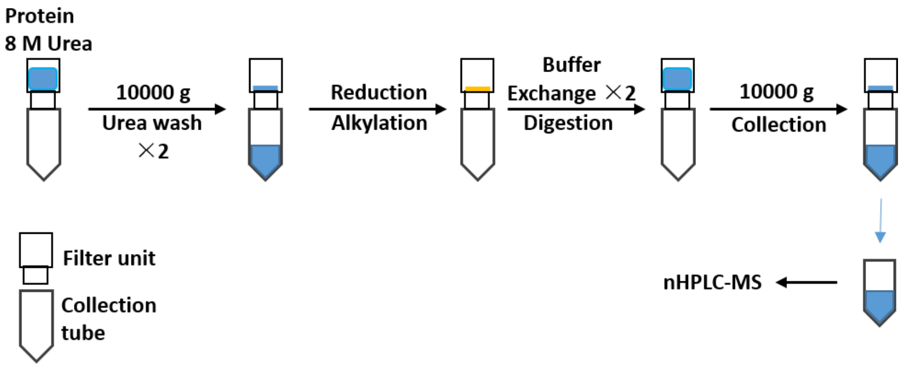

Supplementary Figure 2. Workflow for filter-aided sample preparation (FASP).

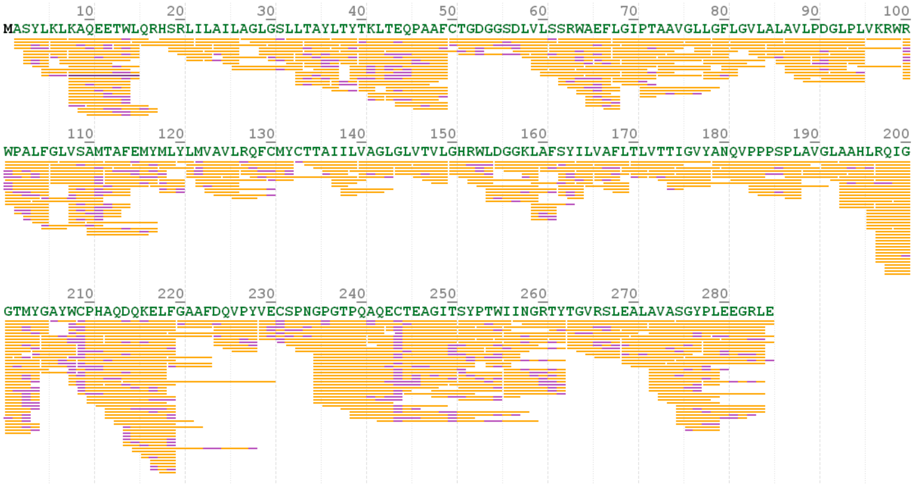

Supplementary Figure 3. MS sequence coverage of VKOR in bottom-up proteomics as determined by Biologic™ software (100%).

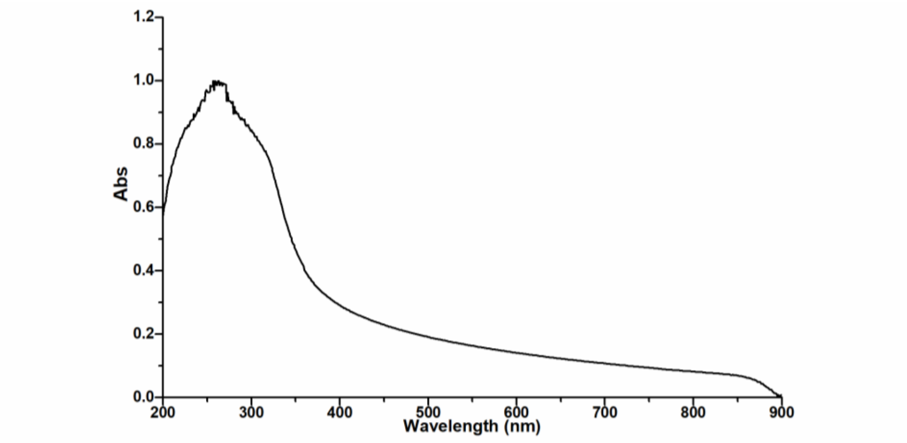

Supplementary Figure 4. UV-Vis absorption spectrum of TiO_2 NPs suspension in water. Source data are provided as a Source Data file.

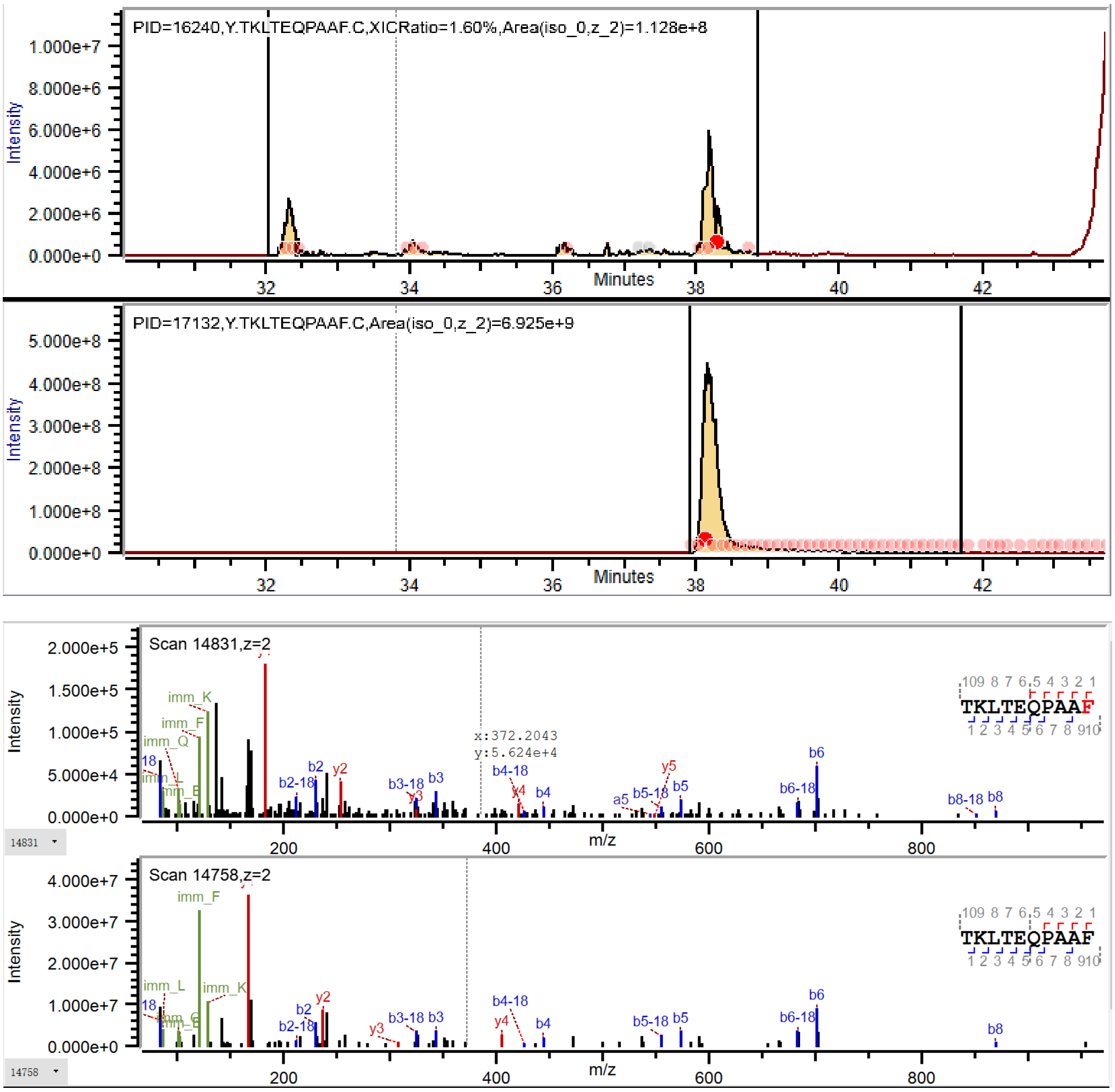

Supplementary Figure 5. EIC and product-ion (MS/MS) spectra of modified (top) and unmodified (bottom) peptide 40-49 from VKOR.

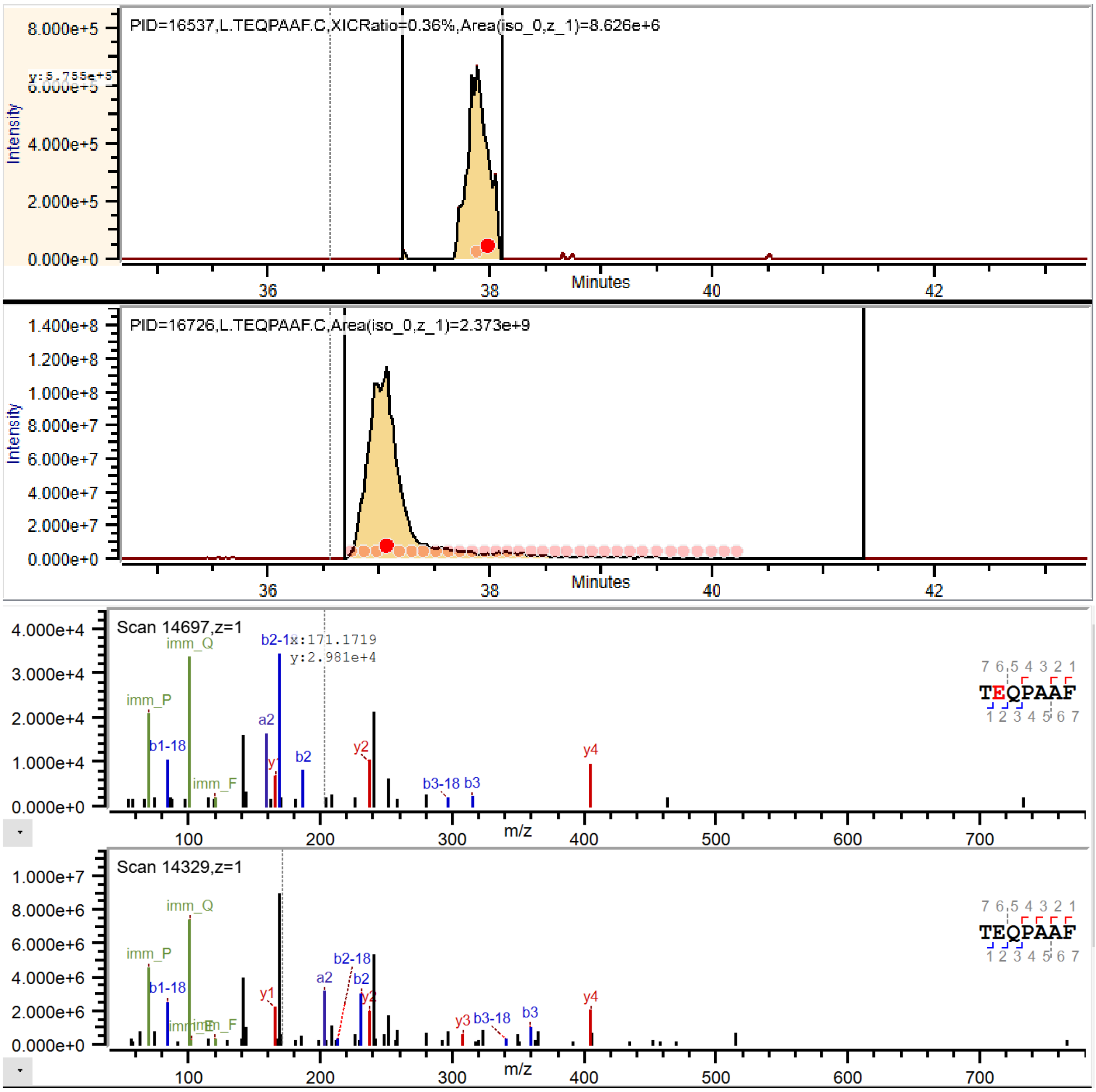

Supplementary Figure 6. EIC and product-ion (MS/MS) spectra of modified (top) and unmodified (bottom) peptide 43-49 from VKOR.

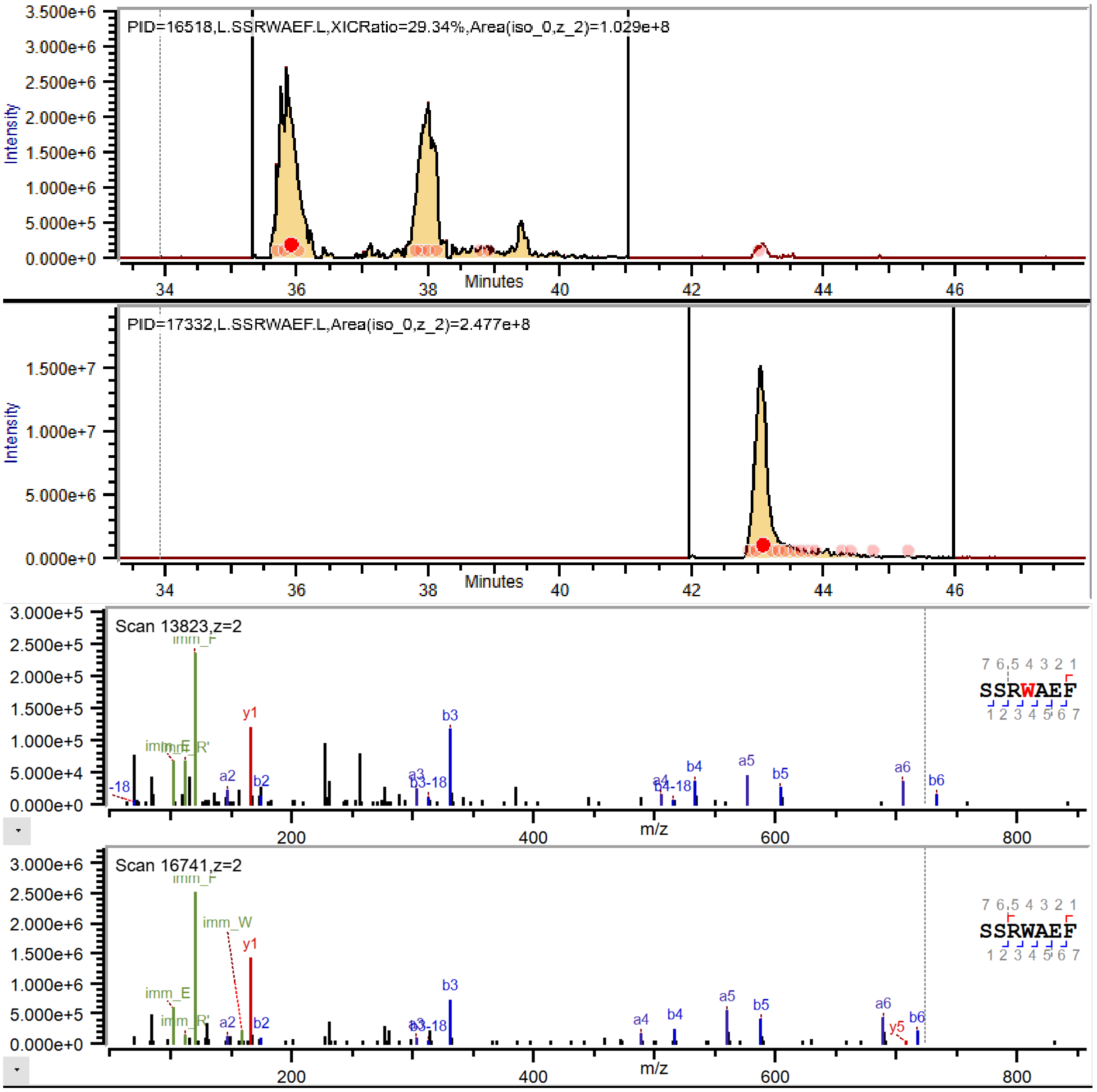

Supplementary Figure 7. EIC and product-ion (MS/MS) spectra of unmodified (top) and modified (bottom) peptide 61-67 from VKOR.

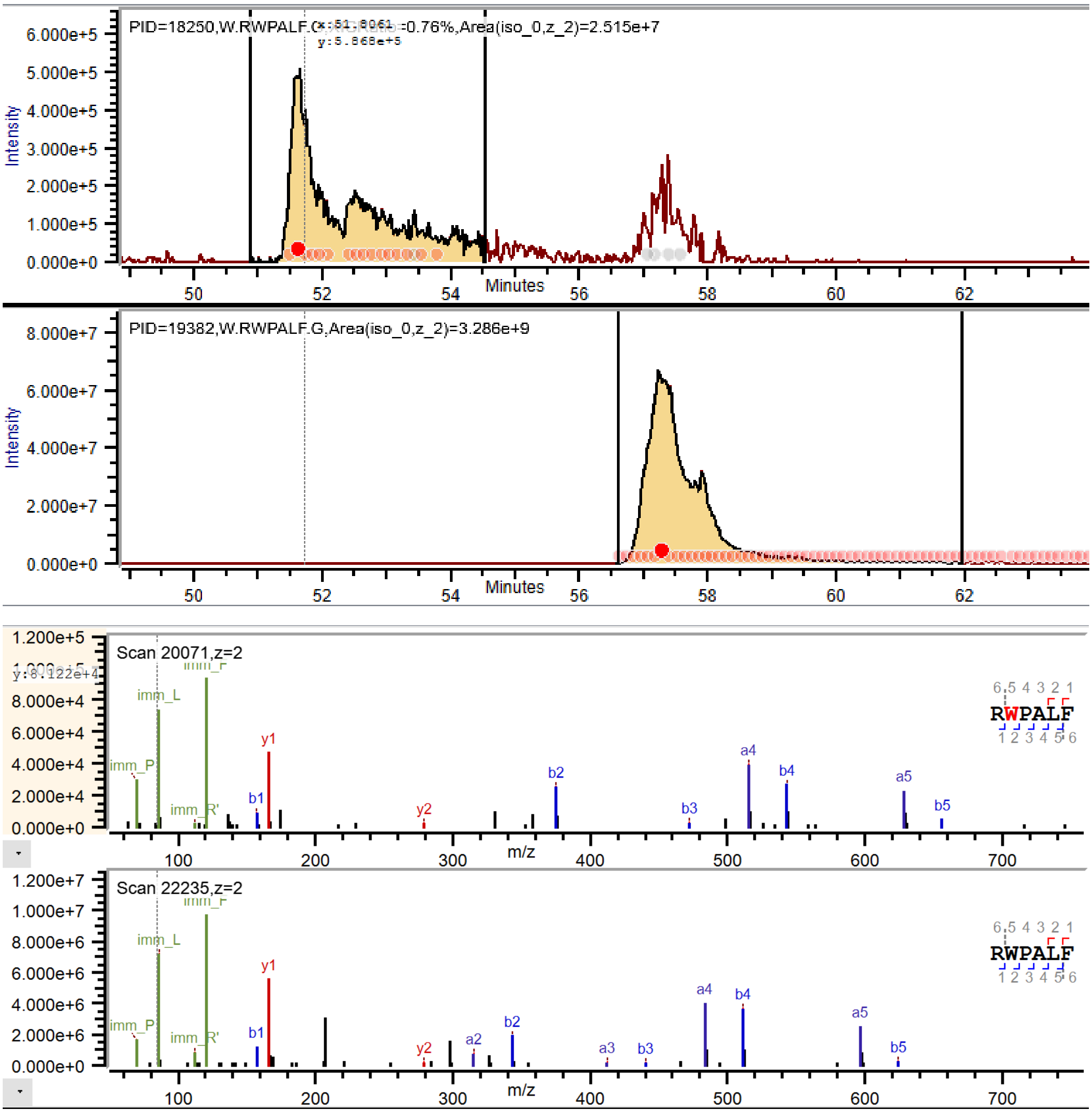

Supplementary Figure 8. EIC and product-ion (MS/MS) spectra of modified (top) and unmodified (bottom) peptide 100-105 from VKOR.

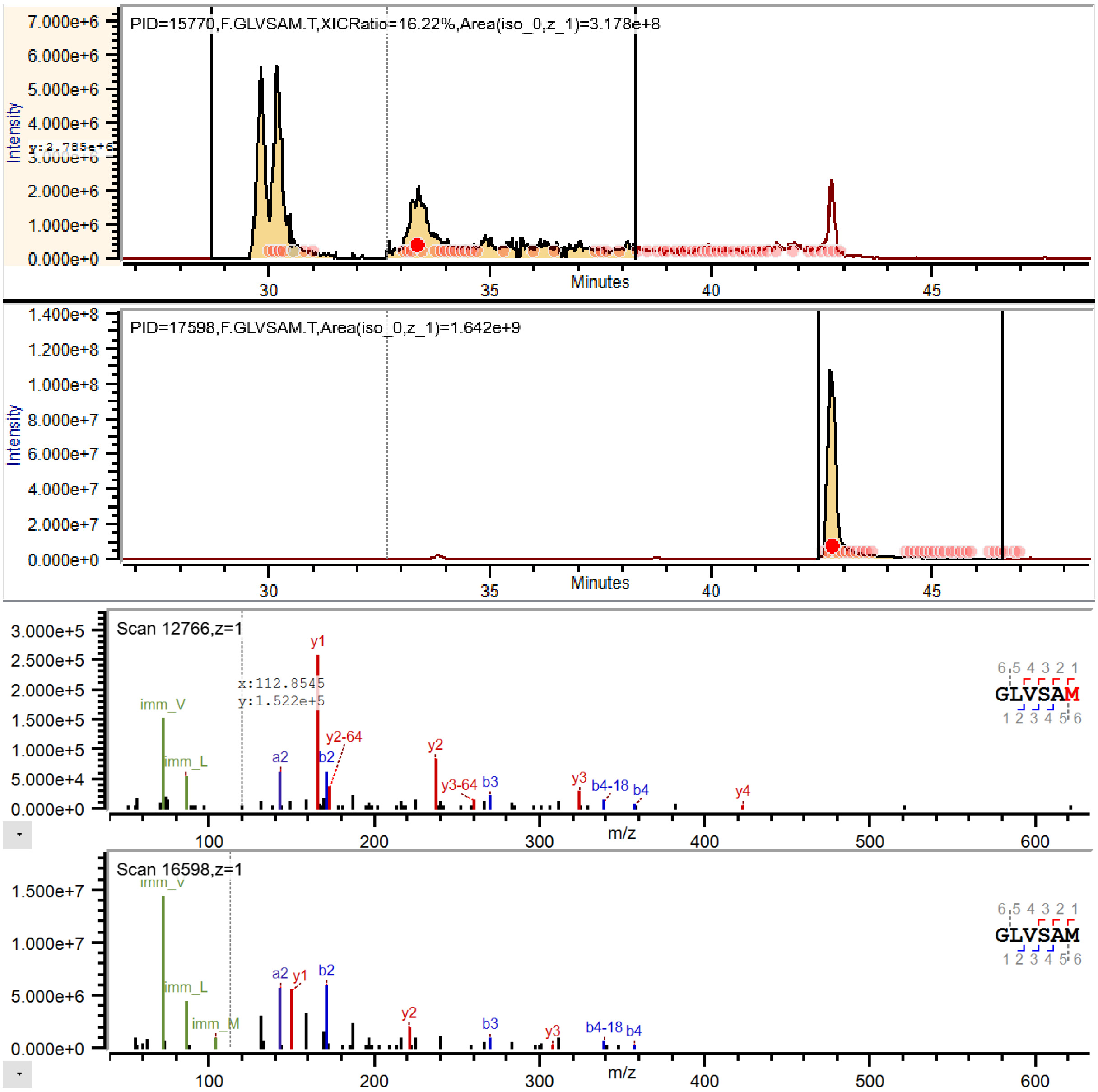

Supplementary Figure 9. EIC and product-ion (MS/MS) spectra of modified (top) and unmodified (bottom) peptide 106-111 from VKOR.

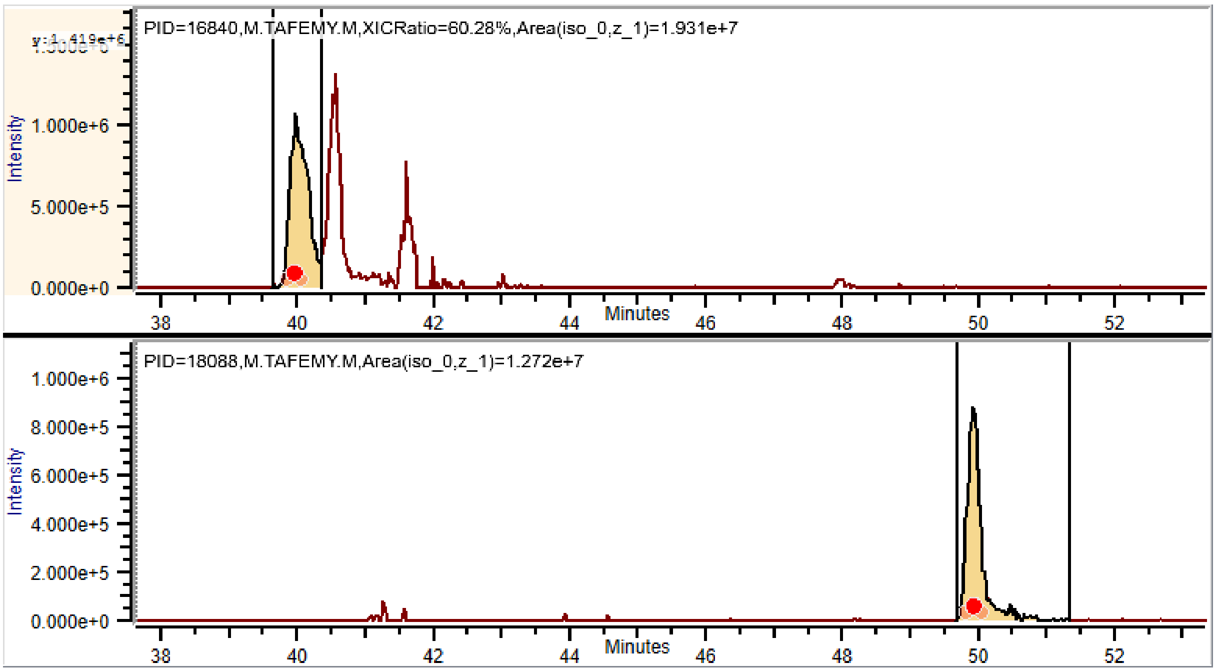

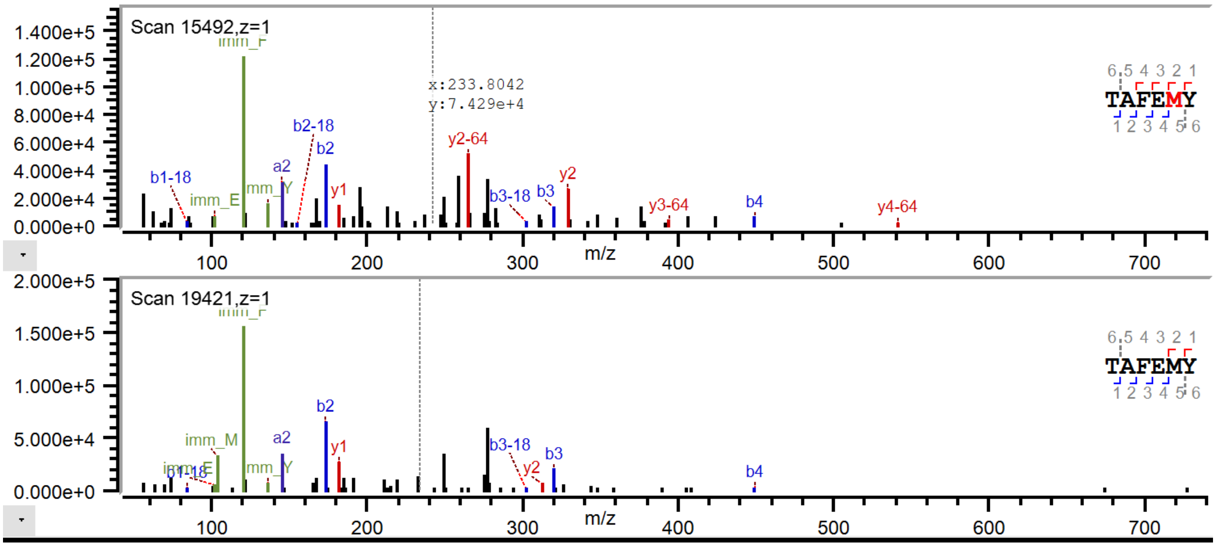

Supplementary Figure 10. EIC and product-ion (MS/MS) spectra of unmodified (top) and modified (bottom) peptide 112-117 from VKOR.

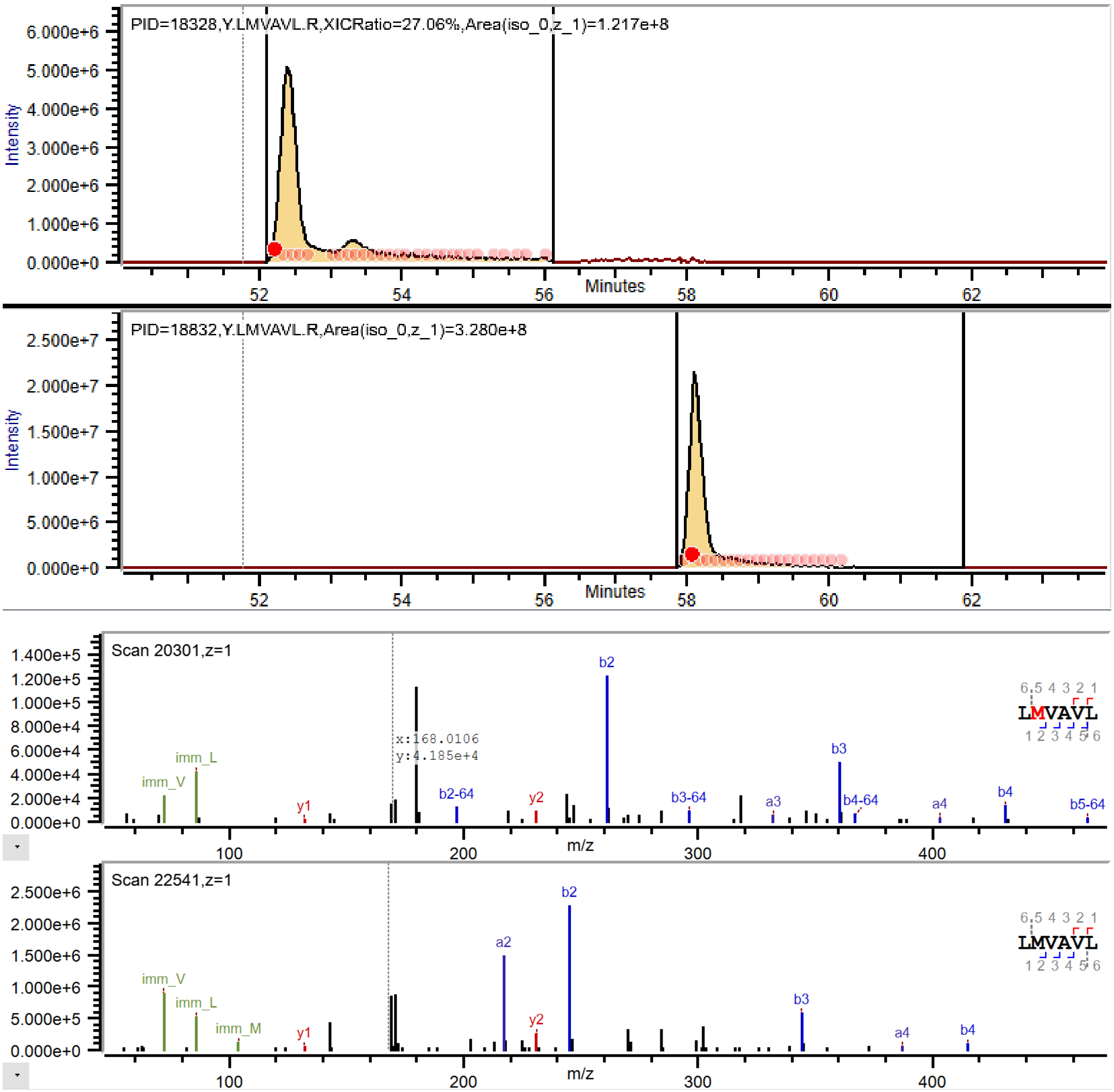

Supplementary Figure 11. EIC and product-ion (MS/MS) spectra of modified (top) and unmodified (bottom) peptide 121-126 from VKOR.

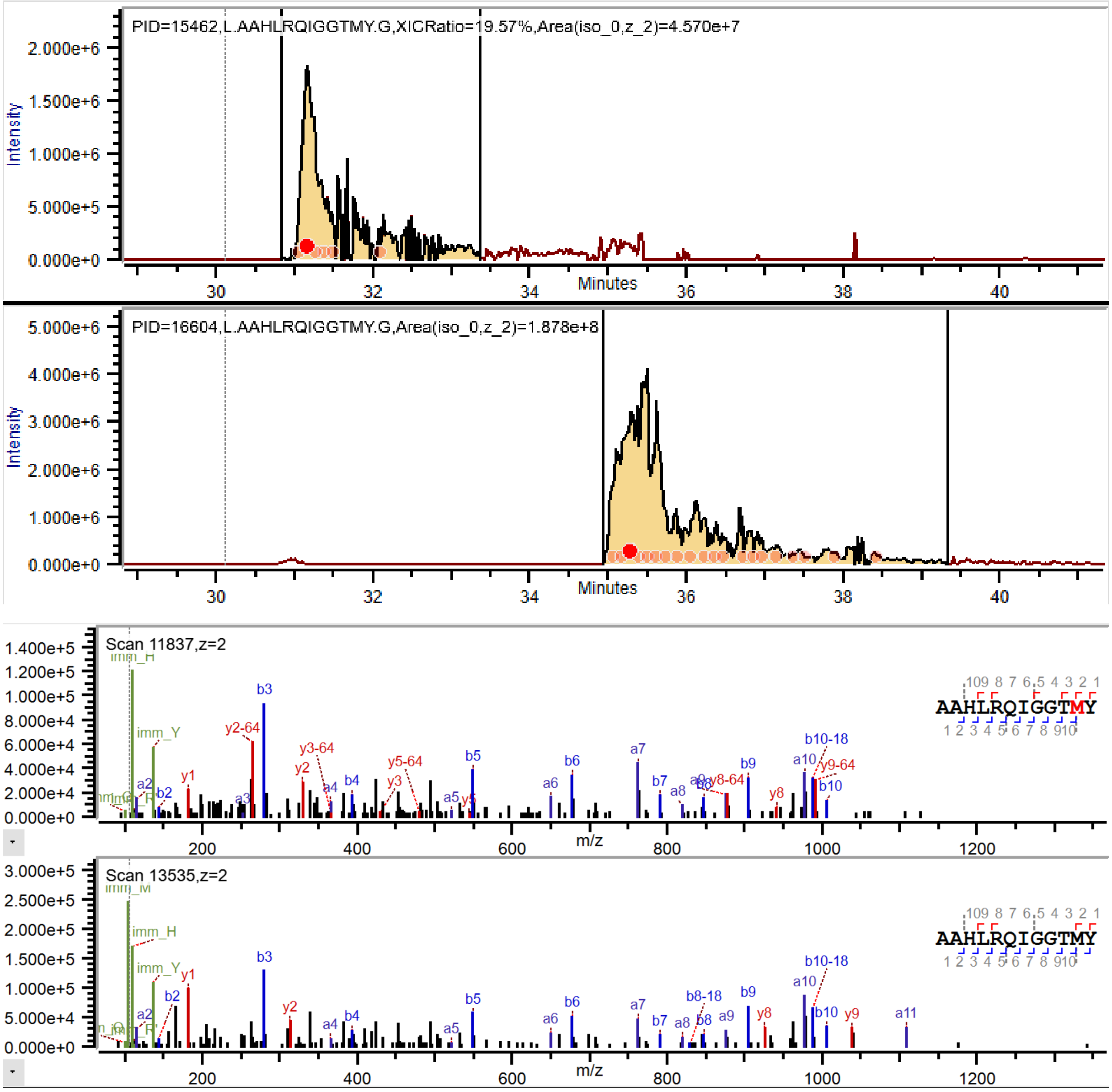

Supplementary Figure 12. EIC and product-ion (MS/MS) spectra of modified (top) and unmodified (bottom) peptide 193-204 from VKOR.

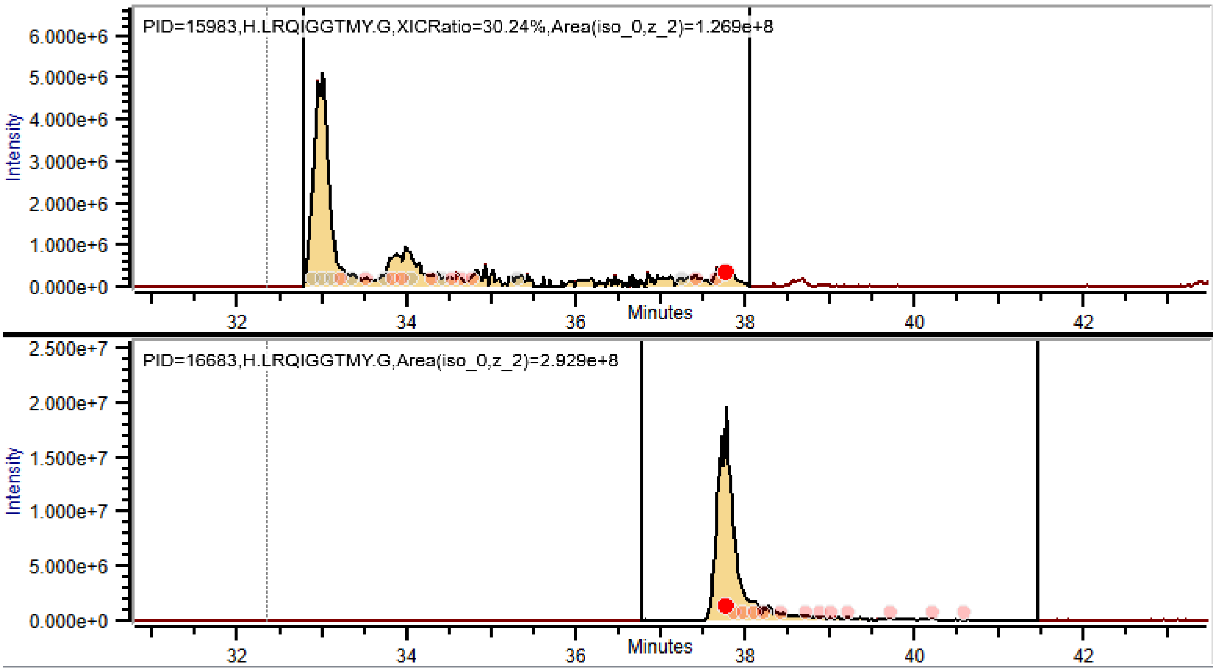

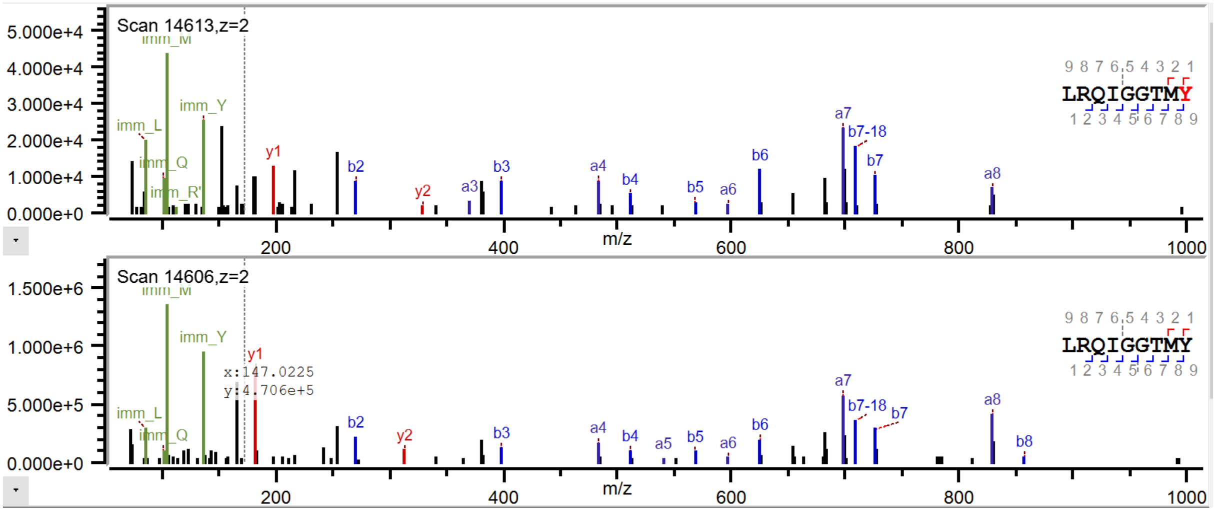

Supplementary Figure 13. EIC and product-ion (MS/MS) spectra of modified (top) and unmodified (bottom) peptide 196-204 from VKOR.

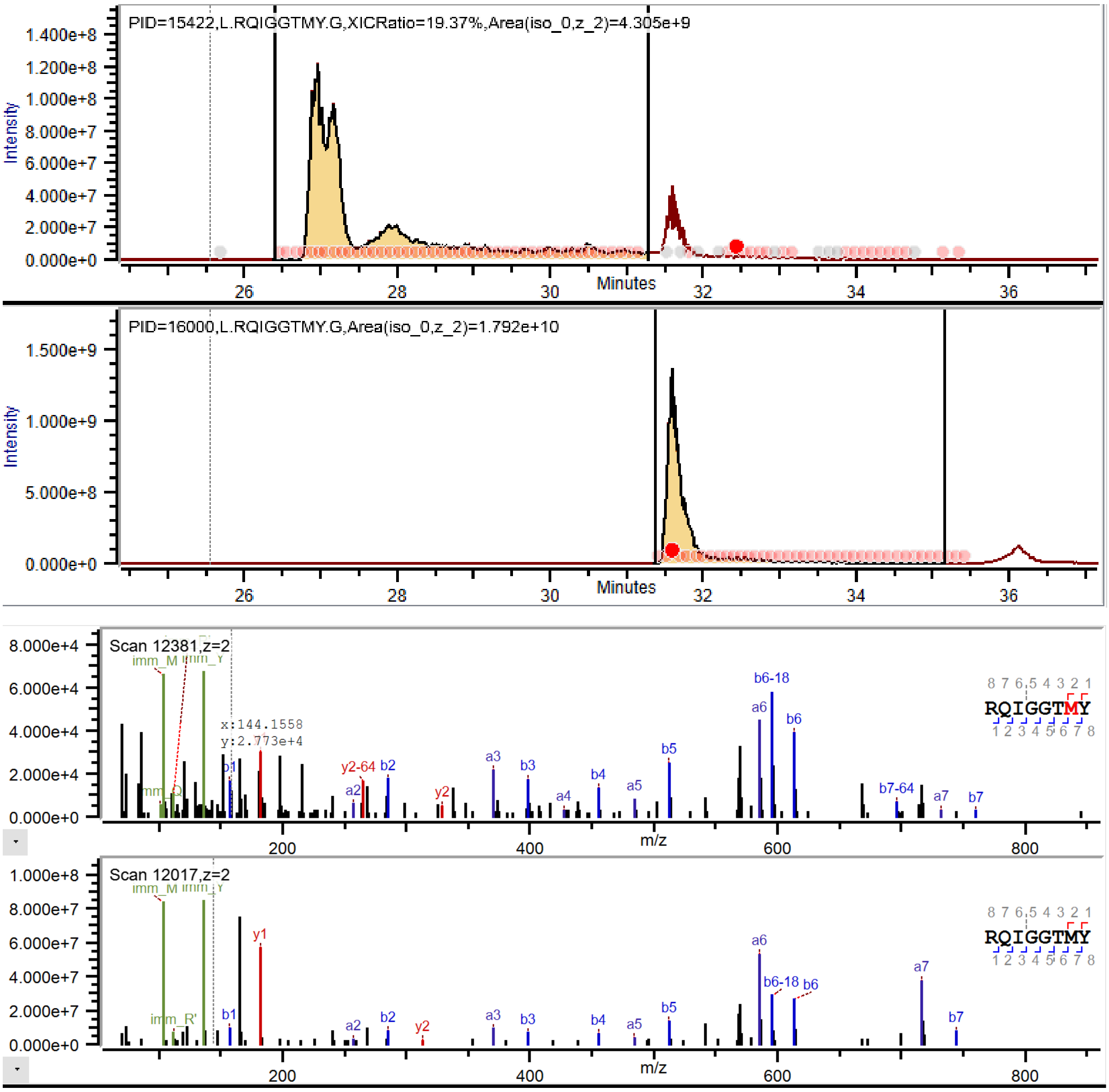

Supplementary Figure 14. EIC and product-ion (MS/MS) spectra of modified (top) and unmodified (bottom) peptide 197-204 from VKOR.

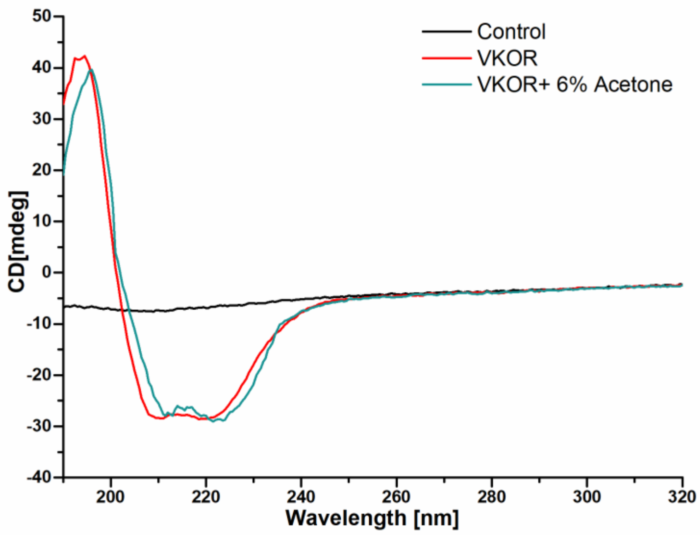

Supplementary Figure 15. Circular dichroism spectrum of membrane protein VKOR. Black line is for the blank. Red line represents 2 μM VKOR in water with 5% DDM detergent. Green line represents 2 μM VKOR in water with 5% DDM detergent and 6% acetone. Source data are provided as a Source Data file.

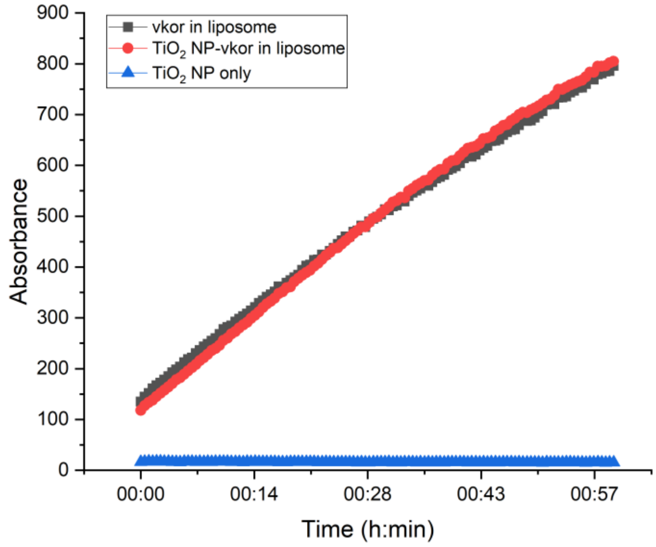

Supplementary Figure 16. VKOR enzymatic activity assay. Y axis represents fluorescence intensity of KH2 with VKOR (5 μM) and TiO_2 NPs. The slope of the curve represents the enzymatic activity of VKOR protein.

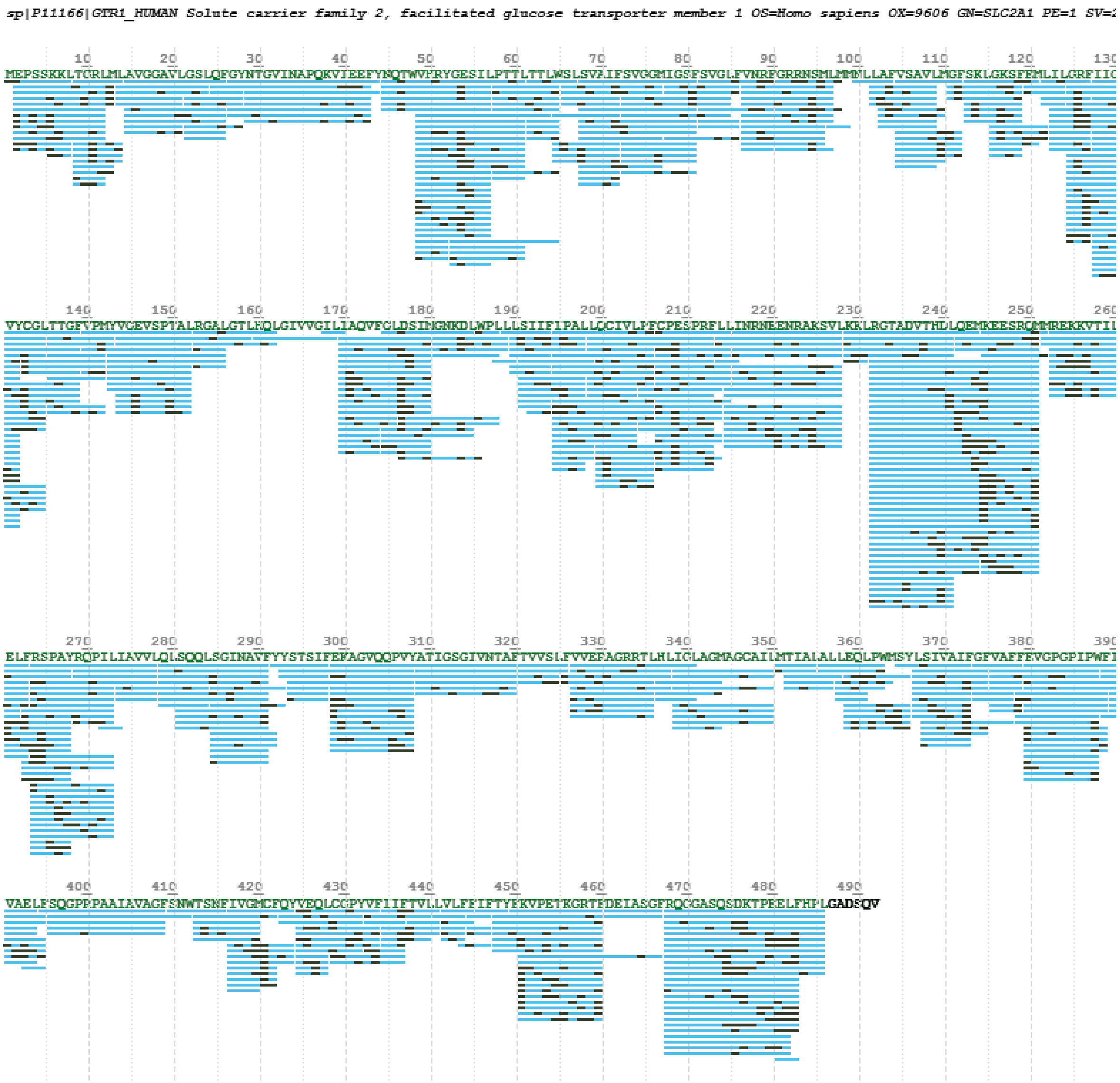

Supplementary Figure 17. MS sequence coverage example of hGLUT1 in bottom-up proteomics obtained by Biologic[TM] software.

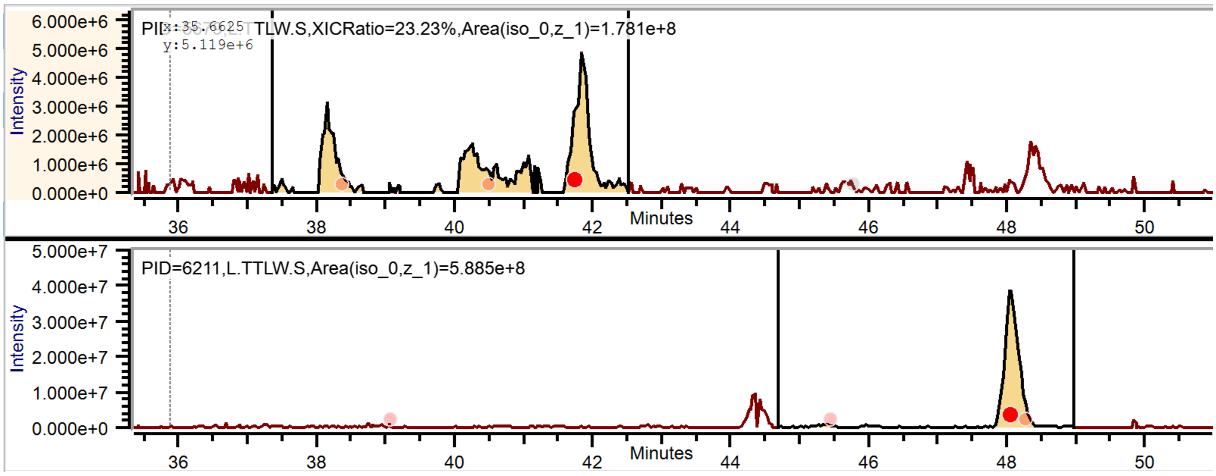

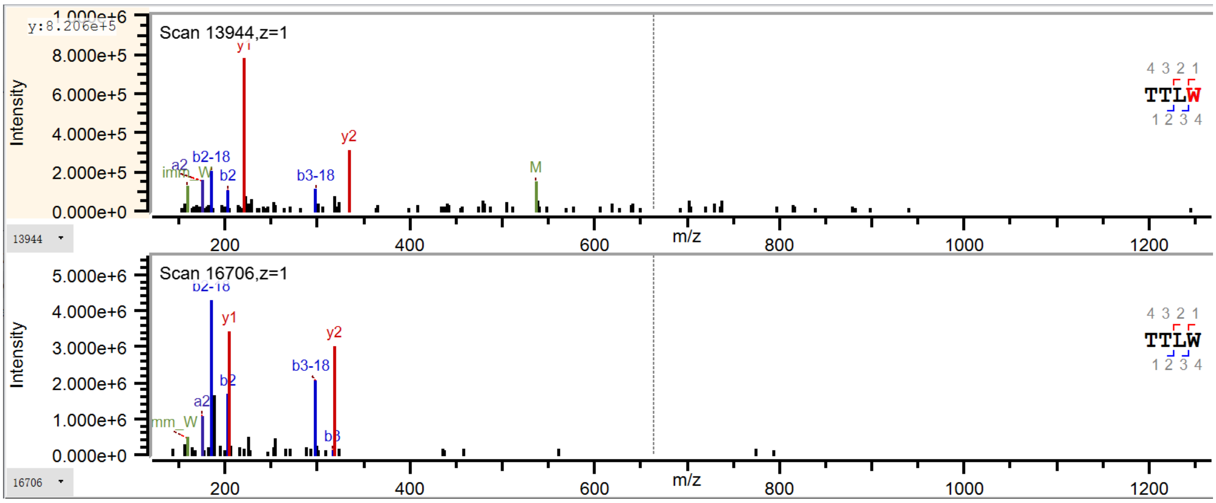

Supplementary Figure 18. (A) EIC and product-ion (MS^2) spectrum of modified and unmodified peptide 62-65 from hGLUT1.

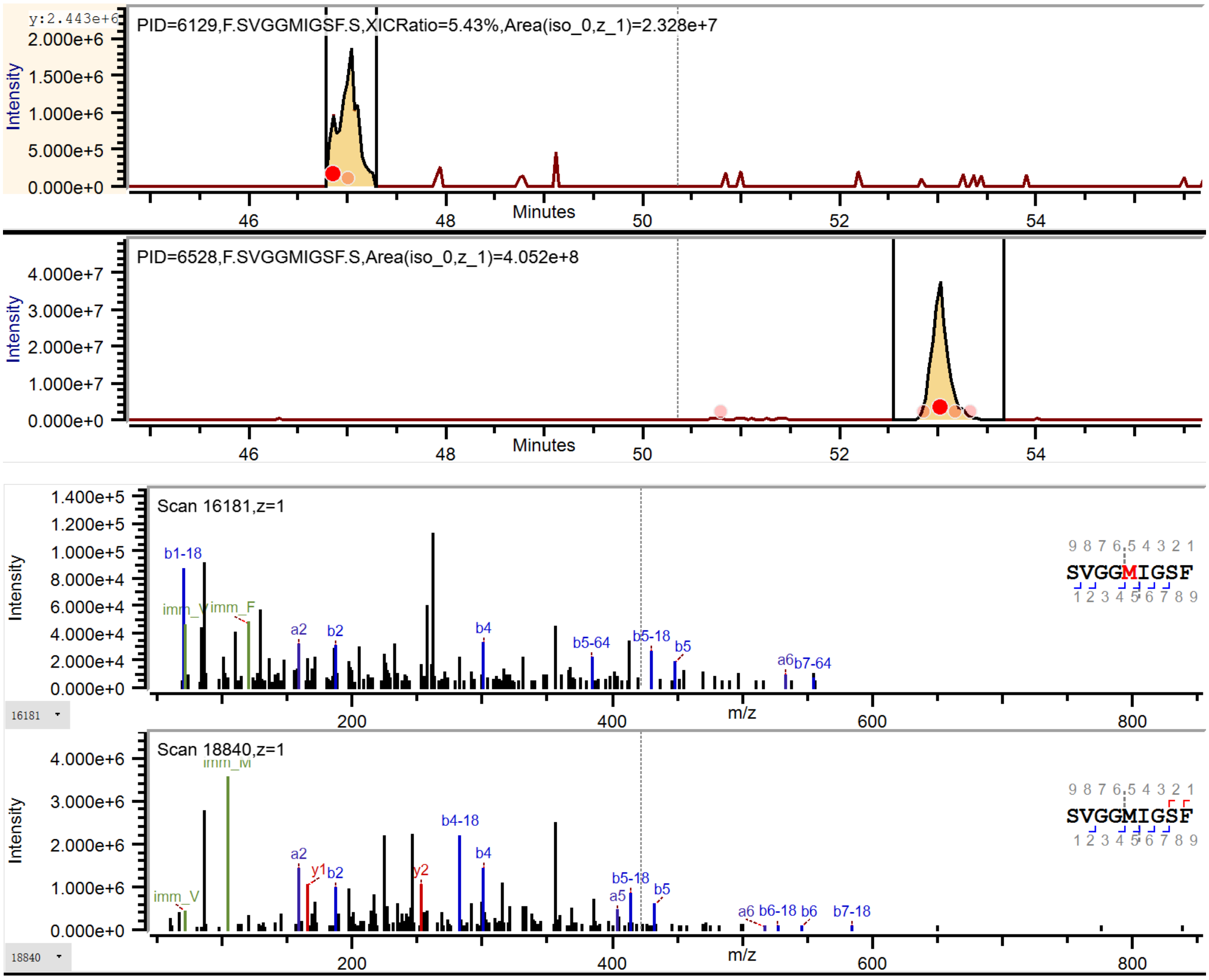

Supplementary Figure 19. (A) EIC and product-ion (MS^2) spectrum of modified and unmodified peptide 73-81 from hGLUT1.

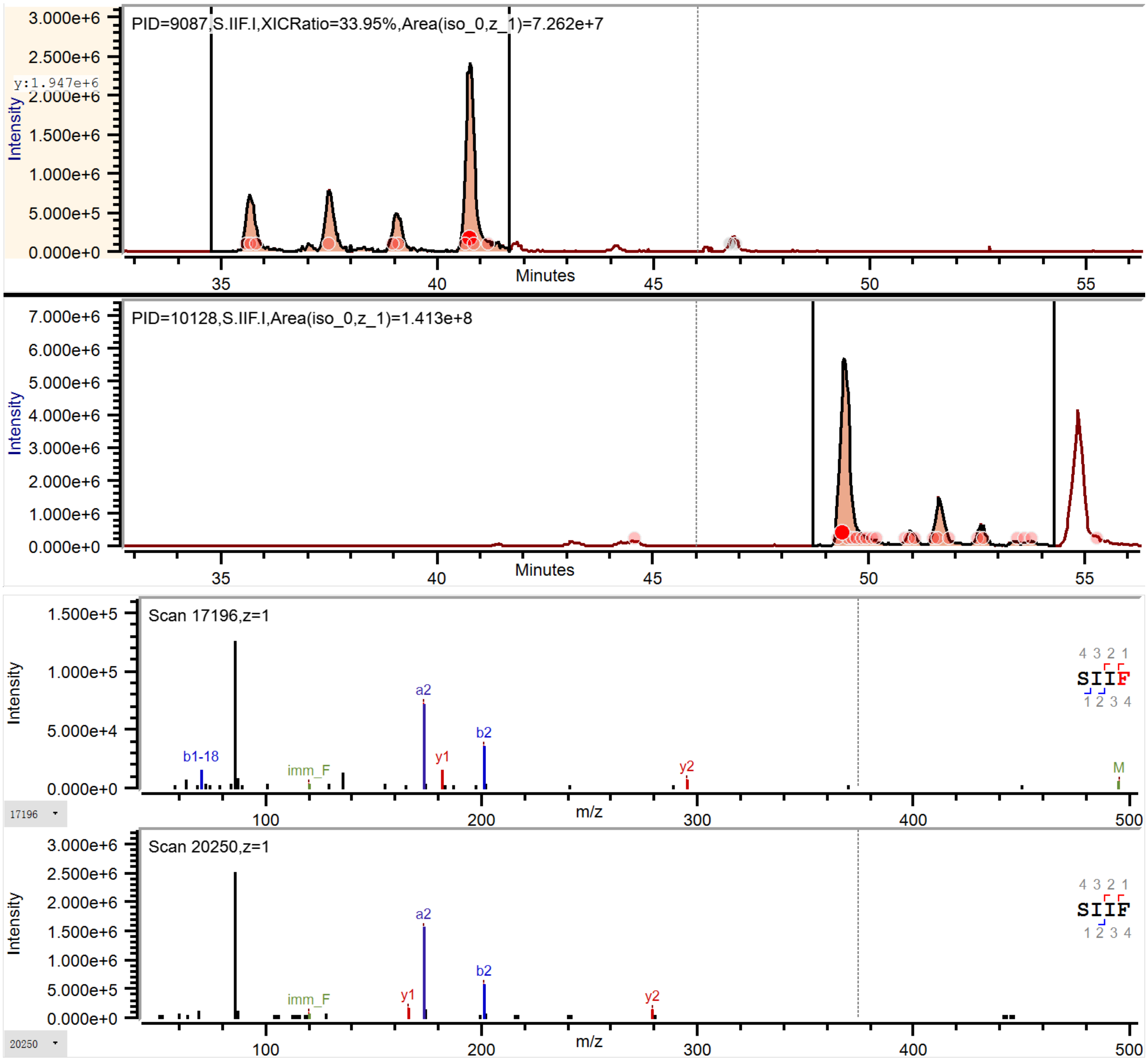

Supplementary Figure 20. (A) EIC and product-ion (MS^2) spectrum of modified and unmodified peptide 191-194 from hGLUT1.

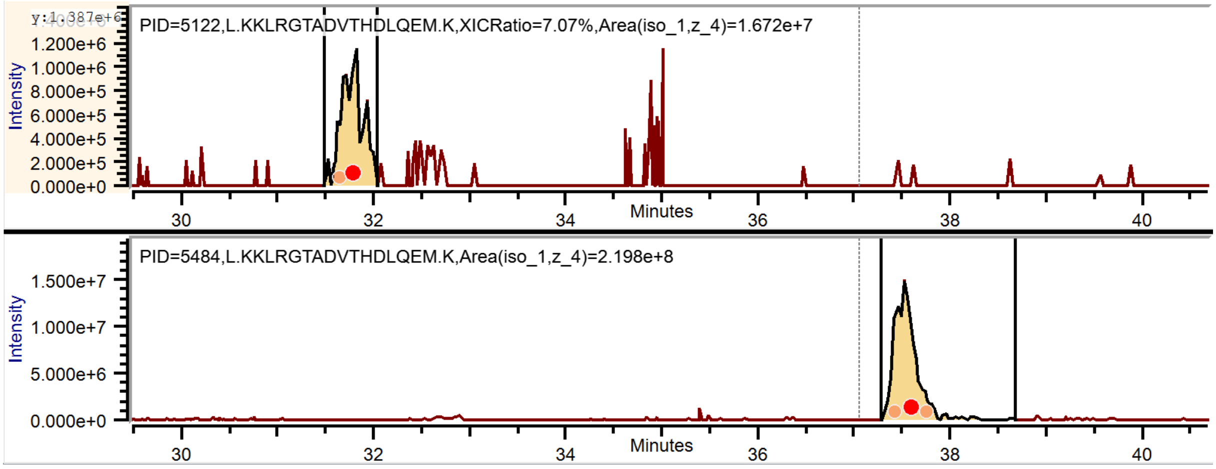

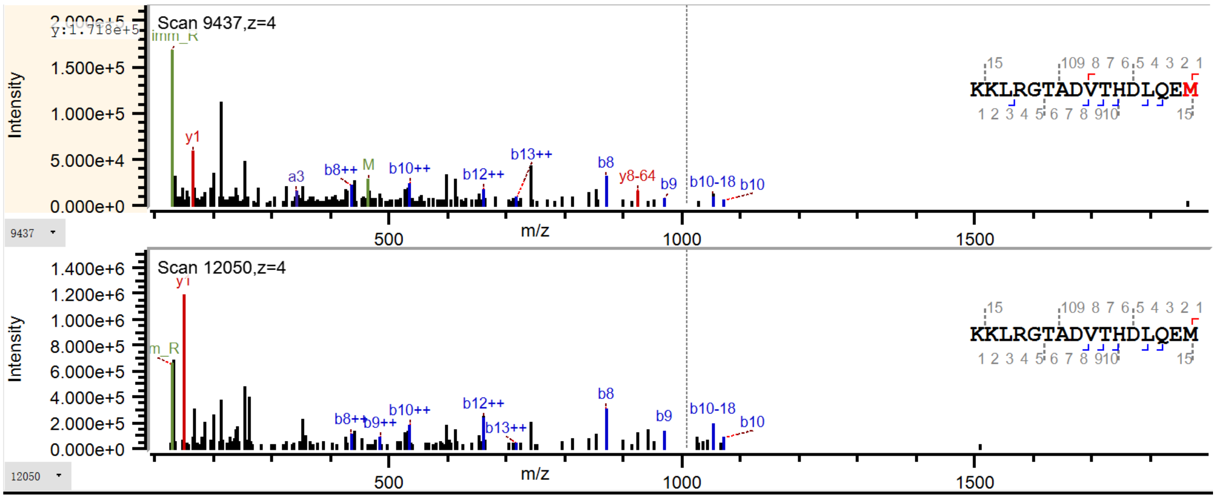

Supplementary Figure 21. (A) EIC and product-ion (MS^2) spectrum of modified and unmodified peptide 229-244 from hGLUT1.

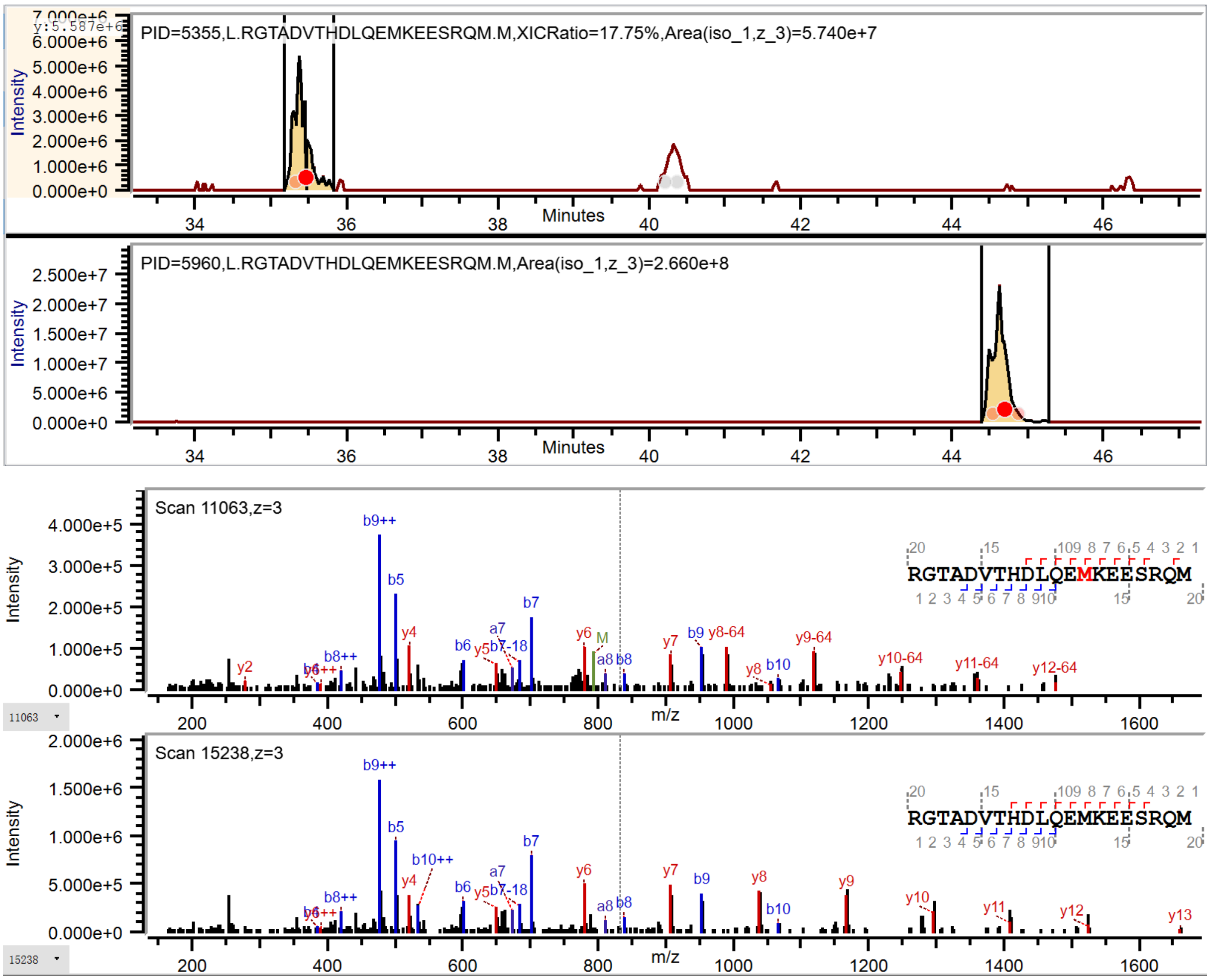

Supplementary Figure 22. (A) EIC and product-ion (MS^2) spectrum of modified and unmodified peptide 232-251 from hGLUT1.

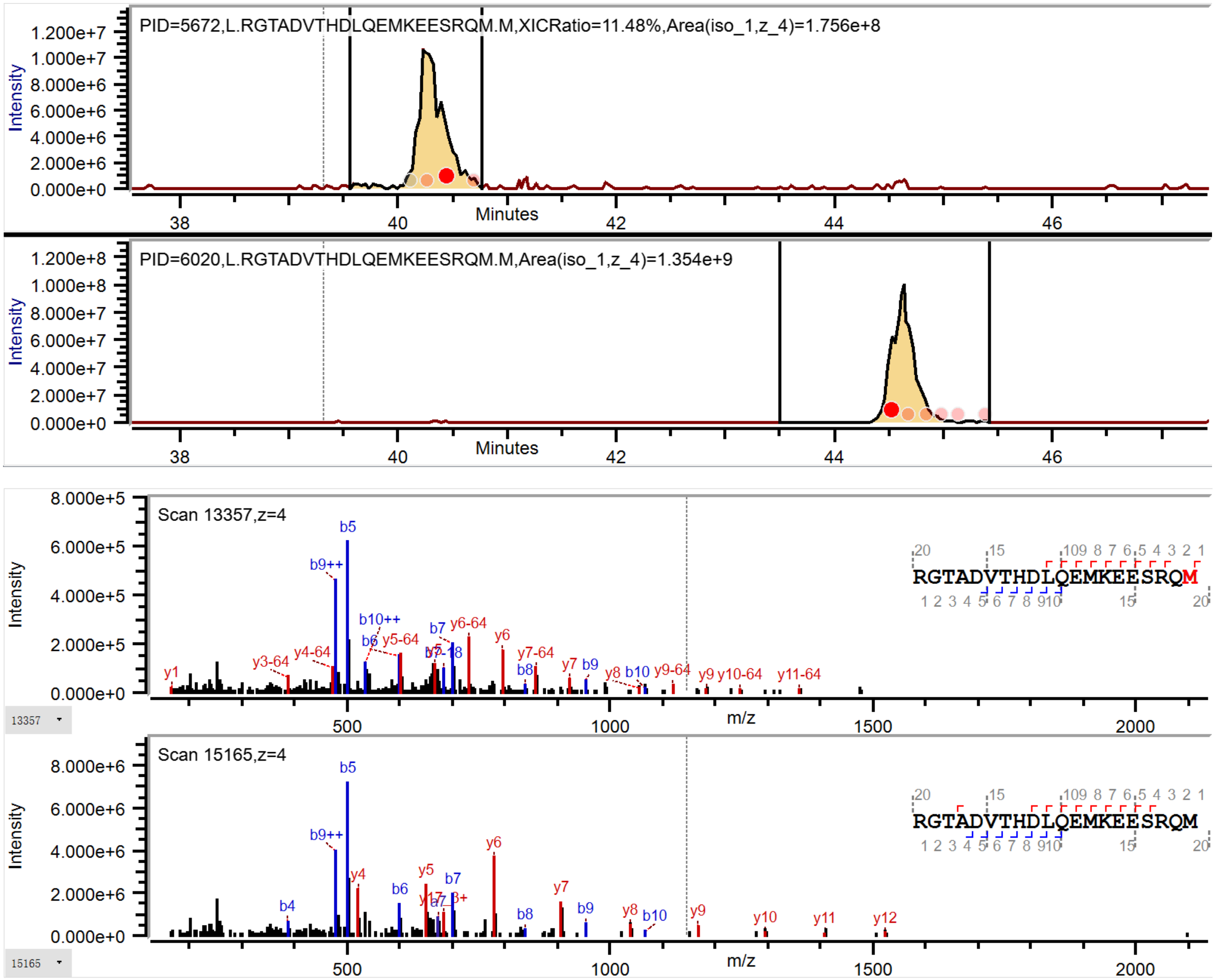

Supplementary Figure 23. (A) EIC and product-ion (MS^2) spectrum of modified and unmodified peptide 232-251 from hGLUT1.

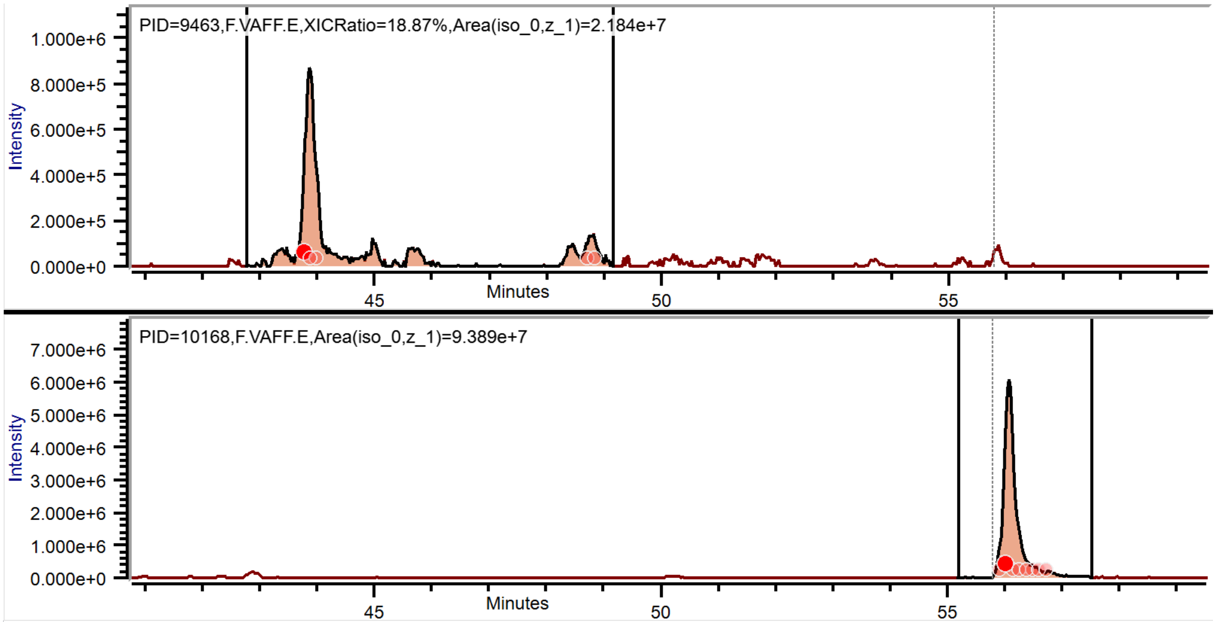

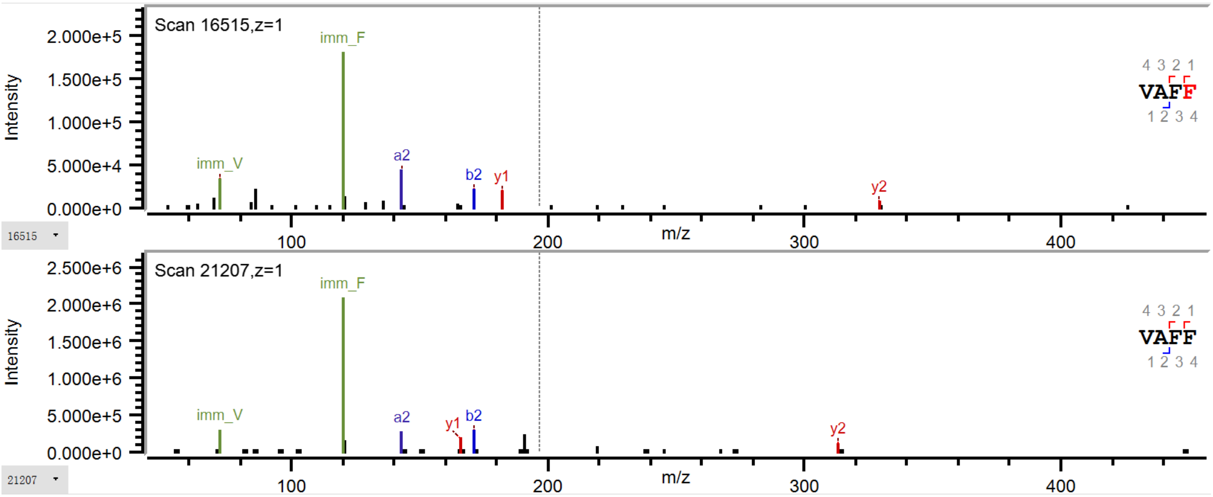

Supplementary Figure 24. (A) EIC and product-ion (MS^2) spectrum of modified and unmodified peptide 376-379 from hGLUT1.

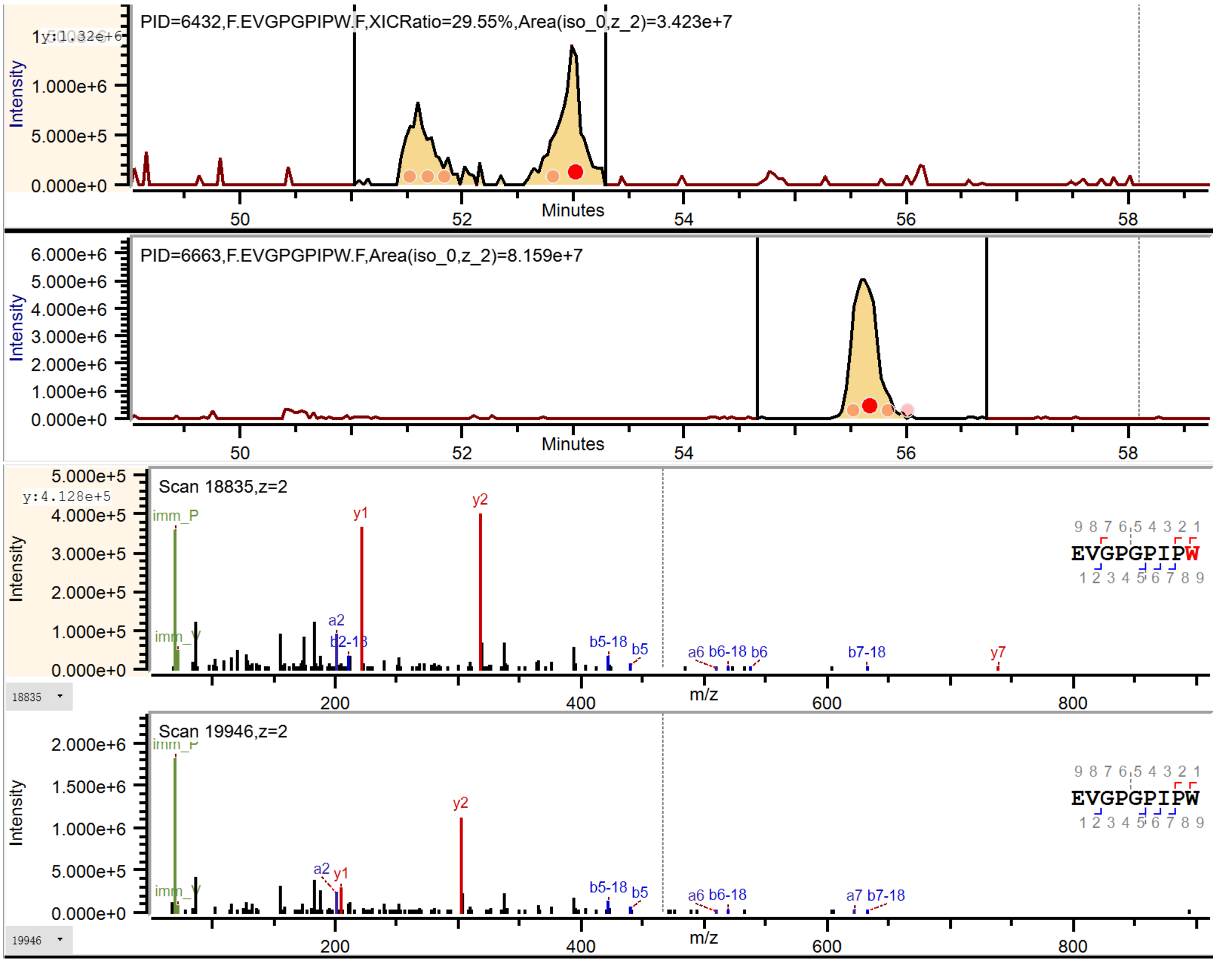

Supplementary Figure 25. (A) EIC and product-ion (MS^2) spectrum of modified and unmodified peptide 380-388 from hGLUT1.

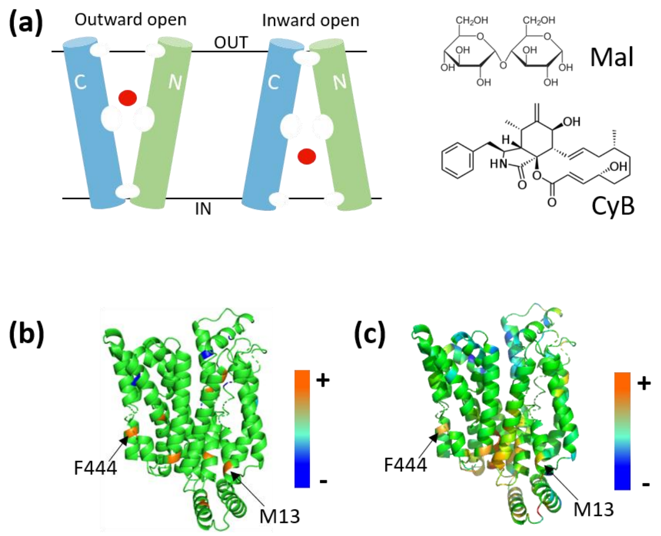

Supplementary Figure 26. SASA change upon ligand binding. (a) Cartoon of outward open and inward open models with binding to Mal and CyB, respectively. Red dot represents the binding ligand. (b) NanoPOMP modification extent difference of [CyB bound state – Mal bound model state] shown on hGLUT1 3D structure. Orange represents increased modification extents. (c) Calculated SASA difference: [CyB bound state – Mal bound model state]. Orange represents higher SASA.

2. Supplementary Tables

Supplementary Table 1. Optimization of several conditions for NanoPOMP. All volumes are in μL.

	0%	2%	4%	5%	6%	8%	10%	13%	15%
**VKOR-liposome**	2.5	2.5	2.5	2.5	2.5	2.5	2.5	2.5	2.5
**TiO_2**	5.0	5.0	5.0	5.0	5.0	5.0	5.0	5.0	5.0
**Tris buffer**	42.5	41.5	40.5	40	39.5	38.5	37.5	36	35
**Acetone**	0	1	2	2.5	3	4	5	6.5	7.5

Supplementary Table 2. NanoPOMP experiments of hGLUT1 in liposome. All volumes are in μL.

	A	B	C	D	E
**GLUT1-liposome**	5.0	5.0	5.0	5.0	5.0
**TiO_2**	5.0	5.0	5.0	5.0	5.0
**Acetone**	0	3	3	3	3
**Tris buffer**	40	37	32	32	32
**Substrates**	--	--	5 (glucose)	5 (Mal)	5 (CyB)

Supplementary Table 3. TiO_2-FPOP of hGLUT1 at the peptide level.

Peptide Number	*m/z* unmod	*m/z* modified	EIC unmod	EIC modified	Fraction modified	Modified residue
**62-65**	520.277	536.271	1.581E8	8.196E7	3.414	W
**73-81**	854.408	870.403	1.758E8	1.859E7	9.559	M
**140-152**	681.852	689.850	2.132E7	9.798E6	31.49	M
**140-143**	509.243	525.238	6.835E7	1.319E8	65.86	M
**144-150**	344.679	322.689	4.674E8	1.248E7	2.601	E
**191-194**	479.286	495.281	6.595E7	9.750E6	12.88	F
**232-244**	491.565	496.895	8.890E7	5.553E7	38.45	M
**232-251**	788.037	793.368	1.055E7	1.672E7	16.31	M
**299-308**	559.796	537.801	1.124E9	4.268E6	0.378	D
**380-388**	476.250	484.248	6.380E7	2.363E7	27.02	W

Supplementary Table 4. NanoPOMP of hGLUT1 (control group: apo state) on peptide level.

Peptide Number	*m/z* unmod	*m/z* modified	EIC unmod	EIC modified	Fraction modified	Modified residue
**13-21**	830.480	846.477	2.711E6	4.731E6	63.57	M
**62-65**	520.276	552.206	2.307E8	5.139E6	1.608	W
**68-72**	530.307	552.303	3.077E7	3.867E6	12.01	F
**73-81**	854.408	870.403	6.875E6	1.298E7	62.94	M
**99-102**	490.269	506.264	3.563E7	7.835E6	18.02	M
**102-104**	350.207	366.202	1.180E8	2.449E7	17.51	F
**105-110**	310.177	318.175	9.440E6	8.756E6	48.35	M
**120-124**	618.352	634.347	1.193E7	6.513E6	35.40	M
**123-127**	303.192	311.189	2.427E8	7.431E6	2.971	R

**140-143**	509.243	525.238	1.793E7	1.800E7	54.00	M
**170-174**	289.170	297.173	5.347E6	2.304E6	12.53	F
**175-180**	635.306	651.300	4.693E7	3.570E6	7.069	M
**191-194**	479.286	495.281	1.165E8	1.070E8	52.13	F
**195-199**	526.360	542.355	5.373E7	1.715E8	23.85	Not sure
**229-244**	461.495	465.493	1.672E7	2.198E8	7.068	M
**232-244**	491.565	496.895	9.340E7	1.464E7	13.54	M
**232-251**	788.037	793.368	5.74E7	2.660E8	17.75	M
**252-260**	373.230	378.502	2.830E7	2.710E7	48.69	M
**261-263**	408.212	424.207	4.378E8	8.409E7	16.36	F
**269-273**	313.703	321.700	1.245E8	2.450E7	16.44	not sure
**287-291**	563.330	579.313	1.250E6	2.232E6	63.11	F
**299-308**	559.796	567.791	2.571E8	6.073E6	2.30	Not sure
**337-341**	276.679	284.676	2.644E7	3.814E6	12.60	I
**376-379**	483.260	499.255	1.011E8	1.636E7	13.82	F
**379-388**	549.785	557.782	7.239E6	6.014E6	26.14	W
**380-388**	476.254	484.251	8.159E7	3.423E7	29.55	W
**389-395**	794.481	838.471	7.456E5	7.474E8	0.100	E
**435-437**	392.254	408.249	2.174E8	11.26E7	33.95	F
**442-444**	378.239	394.234	9.617E7	3.263E7	24.88	F
**445-447**	426.239	442.234	2.486E7	1.323E7	34.68	F
**461-467**	694.341	738.330	1.366E7	3.091E9	0.440	D

Supplementary Table 5. Information of labeled peptide from TiO_2-isopropanol FPOP of hGLUT1.

Peptide	Modification Ratio (%)	Location
62-65	23.23	Extra-membrane
73-81	5.433	TM2
191-194	52.13	TM6
195-199	23.85	TM6
229-244	7.068	Extra-membrane
232-251	5.884	Extra-membrane
299-308	0.658	TM8
309-320	0.551	TM8
376-379	55.39	TM10
379-388	26.14	TM10
380-388	29.55	TM10
389-395	0.1	Extra-membrane
445-447	14.76	TM12
461-467	0.44	Extra-membrane
