# Supplementary material for: Nanoparticles and photochemistry for native-like transmembrane protein footprinting
Source: Nat Commun. 2021 Dec 14;12:7270. doi: 10.1038/s41467-021-27588-8 (PMC8671412; doi:10.1038/s41467-021-27588-8)
Supplement: Supplementary file 2 — Reporting Summary [file 41467_2021_27588_MOESM2_ESM.pdf]

nature portfolio

Corresponding author(s): Michael. L. Gross
Weikai Li

Last updated by author(s): Aug 30, 2021

Reporting Summary

Nature Portfolio wishes to improve the reproducibility of the work that we publish. This form provides structure for consistency and transparency in reporting. For further information on Nature Portfolio policies, see our Editorial Policies and the Editorial Policy Checklist.

Statistics

For all statistical analyses, confirm that the following items are present in the figure legend, table legend, main text, or Methods section.

n/a	Confirmed	
☐	☒	The exact sample size (n) for each experimental group/condition, given as a discrete number and unit of measurement
☐	☒	A statement on whether measurements were taken from distinct samples or whether the same sample was measured repeatedly
☒	☐	The statistical test(s) used AND whether they are one- or two-sided<br>*Only common tests should be described solely by name; describe more complex techniques in the Methods section.*
☒	☐	A description of all covariates tested
☒	☐	A description of any assumptions or corrections, such as tests of normality and adjustment for multiple comparisons
☐	☒	A full description of the statistical parameters including central tendency (e.g. means) or other basic estimates (e.g. regression coefficient) AND variation (e.g. standard deviation) or associated estimates of uncertainty (e.g. confidence intervals)
☒	☐	For null hypothesis testing, the test statistic (e.g. F, t, r) with confidence intervals, effect sizes, degrees of freedom and P value noted<br>*Give P values as exact values whenever suitable.*
☒	☐	For Bayesian analysis, information on the choice of priors and Markov chain Monte Carlo settings
☒	☐	For hierarchical and complex designs, identification of the appropriate level for tests and full reporting of outcomes
☒	☐	Estimates of effect sizes (e.g. Cohen's d, Pearson's r), indicating how they were calculated

Our web collection on statistics for biologists contains articles on many of the points above.

Software and code

Policy information about availability of computer code

Data collection	Thermo Scientific Q Exactive quadrupole orbitrap mass spectrometer
Data analysis	Protein Metrics Suite v3.7-5-g0ce0c0fde2 x64, Thermo Xcalibur 4.0, Pymol (TM) 2.3.2

For manuscripts utilizing custom algorithms or software that are central to the research but not yet described in published literature, software must be made available to editors and reviewers. We strongly encourage code deposition in a community repository (e.g. GitHub). See the Nature Portfolio guidelines for submitting code & software for further information.

Data

Policy information about availability of data

All manuscripts must include a data availability statement. This statement should provide the following information, where applicable:

- Accession codes, unique identifiers, or web links for publicly available datasets
- A description of any restrictions on data availability
- For clinical datasets or third party data, please ensure that the statement adheres to our policy

The mass spectrometry proteomic data generated in this study have been deposited in the ProteomeXchange Consortium via the PRIDE partner repository. MS2 spectra generated are provided in the supporting information. Information of VKOR can be found by 3KP9 [https://www.rcsb.org/structure/3KP9]. Source data are provided with this paper.

Field-specific reporting

Please select the one below that is the best fit for your research. If you are not sure, read the appropriate sections before making your selection.

- [x] Life sciences
- [ ] Behavioural & social sciences
- [ ] Ecological, evolutionary & environmental sciences

For a reference copy of the document with all sections, see nature.com/documents/nr-reporting-summary-flat.pdf

Life sciences study design

All studies must disclose on these points even when the disclosure is negative.

Sample size	No sample-size calculation was performed here. Sample size was determined by number of experiments (triplicates for optimization experiment using model protein VKOR and duplicates for hGLUT1 experiment, that is sufficient for a typical FPOP workflow) needed to illustrate the complete workflow of a NanoPOMP labeling procedure. hGLUT1 sample was limited so two biological repeats were conducted. Two technical replicates (two injections and measurements) were conducted for each individual biologically repeat for hGLUT1. Besides, hGLUT1 contains much more peptides so we tried two measurements to include as much peptides as possible.
Data exclusions	No data exclusion is involved.
Replication	Two independent experiments were conducted for the micrographs. Three independent experiments were performed for the optimization of NanoPOMP by using VKOR. VKOR enzyme activity assay was done with two independent repeats. NanoPOMP experiment for the human membrane protein hGLUT1 were conducted with two independent repeats due to the sample limitation, but each repeat was injected and measured twice. All attempts at replication were successful.
Randomization	Samples were randomized prior to NanoPOMP workflow.
Blinding	No blinding was conducted, as sample class information is needed for the experiment.

Reporting for specific materials, systems and methods

We require information from authors about some types of materials, experimental systems and methods used in many studies. Here, indicate whether each material, system or method listed is relevant to your study. If you are not sure if a list item applies to your research, read the appropriate section before selecting a response.

Materials & experimental systems

n/a	Involved in the study	
[x]	[ ]	Antibodies
[ ]	[x]	Eukaryotic cell lines
[x]	[ ]	Palaeontology and archaeology
[x]	[ ]	Animals and other organisms
[x]	[ ]	Human research participants
[x]	[ ]	Clinical data
[x]	[ ]	Dual use research of concern

Methods

n/a	Involved in the study	
[x]	[ ]	ChIP-seq
[x]	[ ]	Flow cytometry
[x]	[ ]	MRI-based neuroimaging

Eukaryotic cell lines

Policy information about cell lines

Cell line source(s)	HEK293 GnTI– suspension cells (N-acetylglucosaminyltransferase I-negative)
Authentication	This cell line is a gift. We tested the cells viability and live cells number before viruses infection or plasmids transfection. The optimal growth condition for protein expression is above 95% viability and 3 million cell/mL live cell density.
Mycoplasma contamination	Penicillin and streptomycin in cell culture media can prevent mycoplasma contamination. Moreover, we checked cells growth condition using microscopy for each assay.
Commonly misidentified lines (See ICLAC register)	no misidentified lines
